# N-Arylation of amines, amides, imides and sulfonamides with arylboroxines catalyzed by simple copper salt/EtOH system

**DOI:** 10.3762/bjoc.4.40

**Published:** 2008-11-07

**Authors:** Zhang-Guo Zheng, Jun Wen, Na Wang, Bo Wu, Xiao-Qi Yu

**Affiliations:** 1 Key Laboratory of Green Chemistry & Technology (Sichuan University), Ministry of Education, College of Chemistry, Sichuan University, Chengdu 610064, China.

**Keywords:** N-arylation, arylboroxine, copper salt, cross-coupling, ethanol

## Abstract

The coupling of arylboroxines with a variety of amines, amides, imides and sulfonamides catalyzed by a copper salt/EtOH system has been developed. In the absence of a base or additive the corresponding N-arylation products were obtained in moderate to excellent yields.

## Introduction

The copper-mediated N-arylation reaction plays an important role in organic synthesis since the resultant products, arylamines and N-arylheterocyclic compounds, are ubiquitous compounds in pharmaceuticals, crop-protection chemicals and material science [1–4]. In 1997, the copper-mediated heteroatom arylation reaction using arylboronic acids as aryl donors was discovered by Chan, Evans and Lam independently [5–7]. Based on these studies, further improvements to the catalytic variation of organoboron compounds cross coupling have been reported. Among these organoboron compounds, arylboronic acids are the most used aryl donors in the cross-coupling reaction. However, these reactions were carried out with Et_3_N [8–10], pyridine [10–13], or TMEDA [14] as base, or addition of ligand [15]. These procedures usually also used a halogenated hydrocarbon as solvent [8–12]. Moreover, the reaction rates of these reactions were generally slow, even requiring 3 d for completion [5–7].

An attractive alternative to this approach is to develop a simple and efficient catalytic system under mild reaction conditions. Thus, a simple copper salt-catalyzed N-arylation of imides with arylboronic acids in protic solvent system had been developed in our previous reports [16–17]. Similar catalytic systems using non-halogenated hydrocarbon solvents have also been reported by Kantam and Prakash [18–19]. However, these procedures require an atmosphere of air or O_2_ and the use of high temperature, even reflux conditions. It is very dangerous to introduce oxygen or air into a reactor to regenerate the Cu catalyst under reflux condition because of possible explosion and fire hazards especially on large scale. Recently, Kantam et al. reported the N-arylation of imidazoles and amines with arylboronic acids in good yields in methanol at room temperature using copper-exchanged fluorapatite [20]. However, copper-exchanged fluorapatite is a composite salt prepared by a complex, elaborate procedure and has several limitations for the further application.

**Scheme 1:**
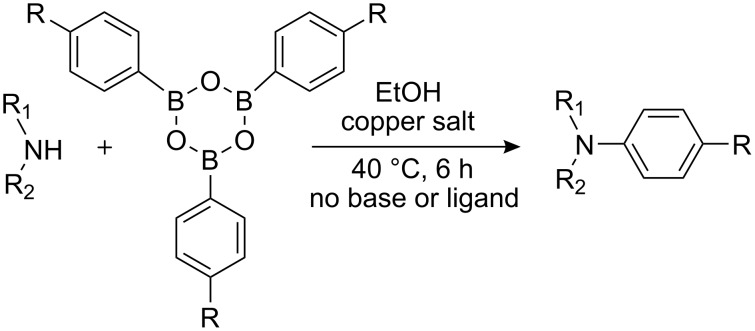
Simple copper salt-catalyzed N-arylation reaction of amines, amides, imides and sulfonamides.

There has been considerable interest recently in the mechanism of the cross-coupling reaction based on boronic acid. The group of Chan has reported the dynamic behavior of boronic acid in the copper salt catalytic system. The results implied that the active arylating agent such as arylboronic acid in the cross-coupling reaction is indeed its anhydride form (boroxine) and not the free acid [21]. This result prompted us to study arylboroxines as aryl donors instead of arylboronic acids in the cross-coupling reaction, since arylboroxine is more active and may remarkably accelerate the reaction. In this paper we found that N-arylation reaction can be more efficiently promoted under milder reaction conditions when an arylboroxine was used. After the reaction conditions had been optimized, a more general and efficient catalytic system for the cross-coupling reaction of N-arylation was developed in presence of only simple copper salt (Scheme 1). Furthermore, we expand the substrate scope of this reaction: a variety of amines, amides, imides and sulfonamides with arylboroxine can also participate in this catalytic system to give the corresponding N-arylation products in moderate to excellent yields. To the best of our knowledge, N-arylation of NH-containing substrates with arylboroxine conducted in protic solvent only with the use of copper salts has not been explored previously.

## Results and Discussion

**Scheme 2:**
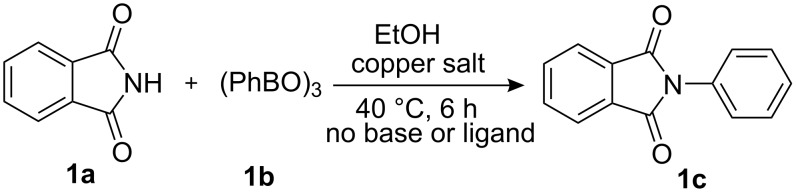
N-arylation reaction of phthalimide with arylboroxine.

Firstly, we chose phthalimide and phenylboroxine as model substrates to optimize the catalytic conditions (copper source, temperature, solvent, amount of catalyst) to achieve the best results in the cross-coupling reactions (Scheme 2). Several simple copper salts were tested as copper sources to promote the coupling reaction with methanol as solvent. As shown in Table 1, most copper salts (20 mol %) that were used gave the desired products in high yields (Table 1, entries 1–10), and the counterion did not play a significant role. This result is much better than previously reported [10] for a similar catalytic system. However, the time required to accomplish this coupling reaction is quite different with these copper salts. In the case of Cu(OTf)_2_, which was chosen as copper source, this coupling reaction took only 6 h to accomplish.

**Table 1:** Optimization of the copper salt for the coupling reaction^a^.

Entry	Copper Salt	Time^b^ (h)	Yield^c^ (%)

1	CuCO_3_·Cu(OH)_2_·H_2_O	24	93
2	Cu(NO_3_)_2_·2H_2_O	11	86
3	Cu(ClO_4_)_2_·7H_2_O	6	75
4	CuSO_4_·5H_2_O	48	90
5	Cu(OAc)_2_·H_2_O	48	86
6	CuCl_2_·2H_2_O	12	84
7	Cu(OTf)_2_	6	98
8	CuCl	8	98
9	CuBr	20	98
10	CuI	20	98

^a^Reaction conditions: **1a** (0.5 mmol), **1b** (0.5 mmol), copper salt (0.1 mmol), methanol (5 ml); temperature 40 °C; ^b^Monitored by TLC; ^c^Isolated yield, purity confirmed by MS and ^1^H NMR.

Reaction temperature plays a crucial role in the cross-coupling reaction. It is reported that reaction time may sometimes be dramatically affected by changing the reaction temperature, which creates opportunities for the activation of the catalytic system. We found that increasing the temperature remarkably accelerated the reaction (Table 2, entries 1–7). A high yield was obtained when the coupling reaction was carried out in methanol at 40 °C within 6 h (Table 2, entry 5). The coupling reaction at 0 °C for almost 2 d gave the same yield of desired product. However, for this cross-coupling reaction, higher temperature was unfavorable as more by-product biphenyl was obtained.

**Table 2:** Optimization of the temperature for the coupling reaction^a^.

Entry	Temperature (°C)	Time^b^ (h)	Yield^c^ (%)

1	0	48	98
2	10	30	98
3	20	20	98
4	30	15	98
5	40	6	98
6	50	18	98
7	60	72	43

^a^Reaction conditions: **1a** (0.5 mmol), **1b** (0.5 mmol), Cu(OTf)_2_ (0.1 mmol), methanol (5 ml); ^b^Monitored by TLC; ^c^Isolated yield, purity confirmed by MS and ^1^H NMR.

The effect of solvent on chemical yield was also examined (Table 3, entries 1–9). We firstly selected PhCH_3_, CH_2_Cl_2_, CH_3_CN as reaction solvents (Table 3, entries 4–6). None of desired products was obtained in the catalytic system of a simple copper salt. However, when protic solvents such as CH_3_OH and EtOH were employed (Table 3, entries 1 and 2), the desired product was obtained in almost quantitative yields in the simple copper salt system. Being of lower toxicity, easier to process and environmentally benign, EtOH was chosen as reaction medium for this coupling reaction.

**Table 3:** Optimization of the solvent for the coupling reaction^a^.

Entry	Solvent (5 ml)	Time^b^ (h)	Yield^c^ (%)

1	CH_3_OH	6	98
2	EtOH	6	98
3	CH_3_COOEt	12	90
4	CH_3_CN	>48	trace
5	PhCH_3_	>48	trace
6	CH_2_Cl_2_	>48	trace
7	THF	10	36
8	DMSO	23	44
9	DMF	5	38

^a^Reaction conditions: **1a** (0.5 mmol), **1b** (0.5 mmol), Cu(OTf)_2_ (0.1 mmol); temperature 40 °C; ^b^Monitored by TLC; ^c^Isolated yield, purity confirmed by MS and ^1^H NMR.

The ratio of phthalimide to arylboroxine and the amount of Cu(OTf)_2_ are both important factors for this coupling reaction (Table 4, entries 1–7). We found that when the ratio is more than 1 : 0.5, the arylated product was obtained in almost quantitative yield (Table 4, entries 1–3). Decreasing the ratio to 1 : 0.3, the arylated product was obtained in only 92% yield (Table 4, entry 4). A decrease of the amount of Cu(OTf)_2_ loading from 20% to 10% had hardly any effect (Table 4, entries 3 and 5), but it took more time to accomplish the reaction when 5 mol % Cu(OTf)_2_ was used (Table 4, entry 6). Decreasing the amount to 2% (Table 4, entry 7), the product was obtained in only 68% within 48 h.

**Table 4:** Optimization of the ratio for the coupling reaction^a^.

Entry	Ratio (**1a : 1b**)	Cu(OTf)_2_ (mol %)	Yield^b^ (%)

1	1 : 1	20	98
2	1 : 0.75	20	98
3	1 : 0.5	20	98
4	1 : 0.3	20	92
5	1 : 0.5	10	98
6	1 : 0.5	5	98^c^
7	1 : 0.5	2	68^d^

^a^Reaction conditions: **1a** (0.5 mmol), EtOH (5 ml); temperature 40 °C, 6 h; ^b^Isolated yield, purity confirmed by MS and ^1^H NMR; ^c^Reaction stirred for 20 h; ^d^Reaction stirred for 48 h.

Good yields of cross-coupled products were also obtained with a variety of substrates bearing methyl- and bromophenylboroxines using phthalimide under our generalized conditions (Table 5, entries 2–3). The results demonstrated that there was little difference between the effect of an electron-rich aryl ring and an electron-deficient aryl ring in this cross-coupling reaction.

**Table 5:** N-Arylation of amines, amides, imides, and sulfonamide with phenylboroxine using copper salt/EtOH system^a^.

Entry	Substrate **a**	Product	Yield^b^ (%)

1	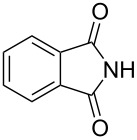	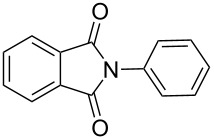 **1c**	98
2	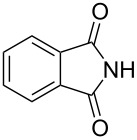	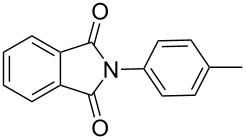 **2c**	96^c^
3	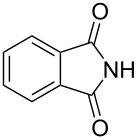	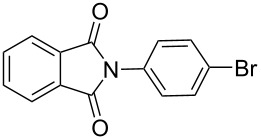 **3c**	98^d^
4	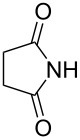	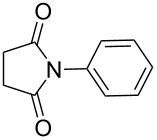 **4c**	98
5	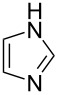	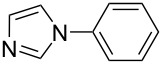 **5c**	92
6	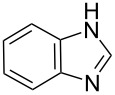	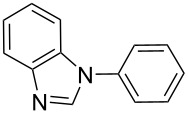 **6c**	99
7	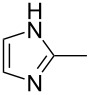	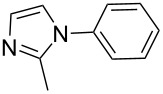 **7c**	93
8	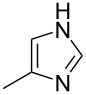	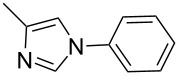 **8c**	92
9	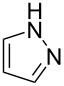	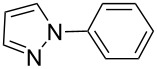 **9c**	40
10	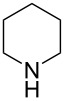	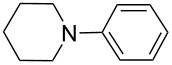 **10c**	42
11	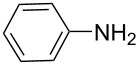	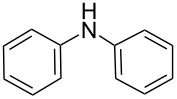 **11c**	62
12	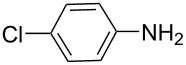	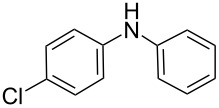 **12c**	60
13	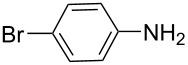	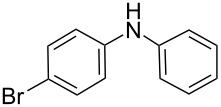 **13c**	58
14	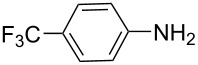	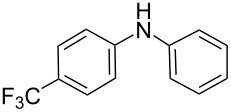 **14c**	55
15	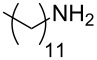	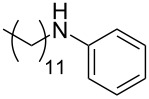 **15c**	63
16	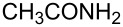	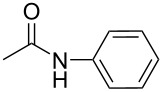 **16c**	40
17	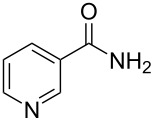	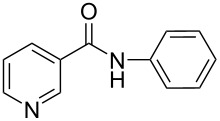 **17c**	56
18	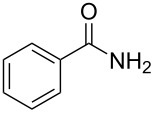	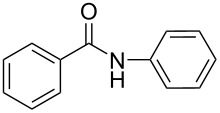 **18c**	41
19	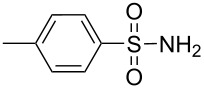	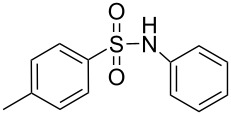 **19c**	30

^a^Reaction conditions: **a** (0.5 mmol), **1b** (0.25 mmol), Cu(OTf)_2_ (0.05 mmol), anhyd EtOH (5 ml), 40 °C; ^b^Isolated yield, purity confirmed by MS and ^1^H NMR; ^c^**2b** (4-CH_3_PhBO)_3_ (0.25 mmol); ^d^**3b** (4-BrPhBO)_3_ (0.25 mmol).

In an endeavor to expand the scope of the above methodology, the catalytic system was also applied to imides, amines, amides and sulfonamides. Such coupling was found to give the desired N-arylation products in moderate yields, as shown in Table 5, except for sulfonamide, which afforded the corresponding products in lower yield (Table 5, entry 19). A series of substituted imidazoles (Table 5, entries 5–8) and imides (Table 5, entries 1–4) were coupled with arylboroxine under the generalized reaction conditions to afford the corresponding products in excellent yields, which are comparable to the literature values using a similar catalyst [19]. It is interesting that bis-arylated products were never detected during the course of the coupling reactions, and the result indicated that this catalytic system had high selectivity for different N-nucleophiles, which is consistent with previous work [16].

In general, the reactions are facile, mild and clean for the synthesis of a variety of N-arylated products. Several functional groups, such as Cl-, Br-, CF_3_- groups remain unaffected under the present reaction conditions.

## Conclusion

In summary, a facile and efficient method for the N-arylation of phthalimide with arylboroxine catalyzed by simple copper salt in EtOH was developed in this paper. The catalytic system is base-free, economical, easy to handle and does not need addition of oxygen. Different reaction conditions such as copper salt, temperature, solvent were systematically optimized. The N-arylation reaction of a variety of amines, amides, imides and sulfonamides with arylboroxine can also occur in this catalytic system to give corresponding N-arylation products in moderate to excellent yields.

## Supporting Information

File 1 N-Arylation of amines, amides, imides and sulfonamides with arylboroxine catalyzed by simple copper salt/EtOH system

## References

[1] Ley S V, Thomas A W (2003). Angew Chem, Int Ed.

[2] Lam P Y S, Vincent G, Clark C G, Deudon S, Jadhav P K (2001). Tetrahedron Lett.

[3] Schlummer B, Scholz U (2004). Adv Synth Catal.

[4] Muci A R, Buchwald S L (2002). Top Curr Chem.

[5] Chan D M T, Monaco K L, Wang R-P, Winters M P (1998). Tetrahedron Lett.

[6] Evans D A, Katz J L, West T R (1998). Tetrahedron Lett.

[7] Lam P Y S, Clark C G, Saubern S, Adams J, Winters M P, Chan D M T, Combs A (1998). Tetrahedron Lett.

[8] Lam P Y S, Bonne D, Vincent G, Clark C G, Combs A P (2003). Tetrahedron Lett.

[9] Chernick E T, Ahrens M J, Scheidt K A, Wasielewski M R (2005). J Org Chem.

[10] Singh B K, Appukkuttan P, Claerhout S, Parmar V S, Van der Eycken E (2006). Org Lett.

[11] Rossiter S, Woo C K, Hartzoulakis B, Wishart G, Stanyer L, Labadie J W, Selwood D L (2004). J Comb Chem.

[12] Jacobsen M F, Knudsen M M, Gothelf K V (2006). J Org Chem.

[13] Nishiura K, Urawa Y, Soda S (2004). Adv Synth Catal.

[14] Yue Y, Zheng Z-G, Wu B, Xia C-Q, Yu X-Q (2005). Eur J Org Chem.

[15] Antilla J C, Buchwald S L (2001). Org Lett.

[16] Lan J-B, Chen L, Yu X-Q, You J-S, Xie R-G (2004). Chem Commun.

[17] Lan J-B, Zhang G-L, Yu X-Q, You J-S, Chen L, Yan M, Xie R-G (2004). Synlett.

[18] Kantam M L, Prakash B V, Reddy C V (2005). J Mol Catal A.

[19] Kantam M L, Neelima B, Reddy C V, Neeraja V (2006). J Mol Catal A.

[20] Kantam M L, Venkanna G T, Sridhar C, Sreedhar B, Choudary B M (2006). J Org Chem.

[21] Chan D M T, Monaco K L, Li R, Bonne D, Clark C G, Lam P Y S (2003). Tetrahedron Lett.

