# Supplementary material for: N-Arylation of amines, amides, imides and sulfonamides with arylboroxines catalyzed by simple copper salt/EtOH system
Source: Beilstein J Org Chem. 2008 Nov 7;4:40. doi: 10.3762/bjoc.4.40 (PMC2587945; doi:10.3762/bjoc.4.40)
Supplement: File 1 — N-Arylation of amines, amides, imides and sulfonamides with arylboroxine catalyzed by simple copper salt/EtOH system [file Beilstein_J_Org_Chem-04-40-s001.doc]

N-Arylation of amines, amides, imides and sulfonamides with arylboroxine catalyzed by simple copper salt/EtOH system

Zhang-Guo Zheng, Jun Wen, Na Wang, Bo Wu, Xiao-Qi Yu*

Key Laboratory of Green Chemistry & Technology (Sichuan University), Ministry of Education, College of Chemistry, Sichuan University, Chengdu 610064, China. Fax: +86 28 85415886; E.mail: xqyu@tfol.com

* Corresponding author

**SUPPORTING INFORMATION**

**Experimental Section**

*General information*: Chemicals and solvents were either purchased or purified by standard techniques. ^1^H NMR spectra were recorded on a Bruker AC 400 instrument at room temperature; CDCl_3_ was used as solvent and TMS as internal standard; chemicals shifts (δ) are reported relative to TMS. Mass spectra were obtained using an Agilent 6890 GC-MS apparatus at 70 eV ionization voltage. Electrospray ionization (ESI) mass spectrometry was performed on a Bruker Daltonics Bio TOF mass spectrometer.

**Arylboroxines 1b**–**3b; General Procedure**

A solution of arylboronic acid (40 mmol) in benzene (100 mL) was refluxed for 2 h, during which H_2_O was removed azeotropically. The mixture was concentrated under reduced pressure into ca. 30 mL, and it was cooled to room temperature. The precipitate obtained by filtration was washed five times with hexane to give arylboroxine as white crystals.

**2,4,6-Triphenyl-1,3,5,2,4,6-trioxatriborinane** (**1b**)

^1^H NMR (400 MHz, CDCl_3_, TMS): δ = 7.49–7.52 (m, 6 H), 7.58–7.60 (m, 3 H), 8.23–8.24 (m, 6 H); MS (EI, 70 eV): *m/z* = 312 [M] ^+^.

**2,4,6-Tri-*p*-tolyl-1,3,5,2,4,6-trioxatriborinane** (**2b**)

^1^H NMR (400 MHz, CDCl_3_, TMS): δ = 2.44 (m, 9 H), 7.30–7.32 (m, 6 H), 8.12–8.14 (m, 6 H); ESI-MS: *m/z* = 354 [M]^+^.

**2,4,6-Tris(4-bromophenyl)-1,3,5,2,4,6-trioxatriborinane** (**3b**)

^1^H NMR (400 MHz, CDCl_3_, TMS): δ = 7.64–7.66 (m, 6 H), 8.04–8.06 (m, 6 H); ESI-MS: *m/z* = 383 [M−2Br]^+^.

**Cross-coupling reaction of N-nucleophiles (preparation of 1c**–**19c); General Procedure**

N-nucleophile (0.5 mmol), (ArBO)_3_ (0.25 mmol), Cu(OTf)_2_ (0.018 g, 0.05 mmol) and anhydrous EtOH (5 mL) were placed in a 25 mL vial. The reaction mixture was vigorously stirred under an atmosphere of air at 40 °C for the appropriate time. After completion of the reaction, as monitored by TLC, the reaction mixture was concentrated and the residue was purified by flash column chromatography on silica gel with *n*-hexane-EtOAc (4 : 1) as the eluent to afford the N-arylated product. The identity and purity of the known products was confirmed by GC-MS, ^1^H NMR. And all their data correspond exactly with those published.

**2-Phenylisoindoline-1,3-dione (1c)** [1]

White solid; yield: 109 mg (98%); ^1^H NMR (400 MHz, CDCl_3_, TMS): δ = 7.40–7.46 (m, 3 H), 7.50–7.54 (m, 2 H), 7.79–7.82 (m, 2 H), 7.97 (m, 2 H); MS (EI, 70 eV): *m/z* = 223 [M]^+^.

**2-*p*-Tolylisoindoline-1,3-dione (2c)** [1]

White solid; yield: 114 mg (96%); ^1^H NMR (400 MHz, CDCl_3_, TMS): δ = 2.41 (s, 3 H), 7.31 (m, 4 H), 7.77–7.80 (m, 2 H), 7.94–7.95 (m, 2 H); ESI-MS: *m/z* = 238 [M+H]^+^.

**2-(4-Bromophenyl)isoindoline-1,3-dione (3c)** [2]

Colorless solid; yield: 148 mg (98%); ^1^H NMR (400 MHz, CDCl_3_, TMS): δ = 7.34–7.38 (m, 2 H), 7.62–7.66 (m, 2 H), 7.80–7.81 (m, 2 H), 7.98 (m, 2 H); ESI-MS: *m/z* = 325 [M+Na]^+^.

**1-Phenylpyrrolidine-2,5-dione (4c)** [3]

Colorless solid; yield: 86 mg (98%); ^1^H NMR (400 MHz, CDCl_3_, TMS): δ = 2.90 (m, 4 H), 7.26–7.30 (m, 2 H), 7.40–7.42 (m, 1 H), 7.47–7.51 (m, 2 H); MS (EI, 70 eV): *m/z* = 175 [M]^+^.

**1-Phenyl-1*H*-imidazole (5c)** [4]

White solid; yield: 66 mg (92%); ^1^H NMR (400 MHz, CDCl_3_, TMS): δ = 7.21 (s, 1 H), 7.27–7.29 (m, 1 H), 7.35–7.40 (m, 3 H), 7.47–7.51 (m, 2 H), 7.86 (s, 1 H); MS (EI, 70 eV): *m/z* = 144 [M]^+^.

**1-Phenyl-1*H*-benzo[d]imidazole (6c)** [5]

White solid; yield: 96 mg (99%); ^1^H NMR (400 MHz, CDCl_3_, TMS): δ = 7.32–7.36 (m, 2 H), 7.46–7.61 (m, 6 H), 7.89–7.93 (m, 1 H), 8.13 (s, 1 H); MS (EI, 70 eV): *m/z* = 194 [M]^+^.

**2-Methyl-1-phenyl-1*H*-imidazole (7c)** [6]

White solid; yield: 74 mg (93%); ^1^H NMR (400 MHz, CDCl_3_, TMS): δ = 2.36 (s, 3 H), 7.00–7.03 (m, 2 H), 7.28–7.30 (m, 2 H), 7.40–7.50 (m, 3 H); MS (EI, 70 eV): *m/z* = 158 [M]^+^.

**4-Methyl-1-phenyl-1*H*-imidazole (8c)** [7]

White solid; yield: 73 mg (92%); ^1^H NMR (400 MHz, CDCl_3_, TMS): δ = 2.30 (m, 3 H), 7.01 (s, 1 H), 7.30–7.36 (m, 3 H), 7.46 (m, 2 H), 7.78 (s, 1 H); MS (EI, 70 eV): *m/z* = 158 [M]^+^.

**1-Phenyl-1*H*-pyrazole (9c)** [5]

Colorless oil; yield: 29 mg (40%); ^1^H NMR (400 MHz, CDCl_3_, TMS): δ = 6.47 (m, 1 H), 7.26–7.31 (m, 1 H), 7.44–7.48 (m, 2 H), 7.69–7.73 (m, 3 H), 7.93 (d, 1 H); MS (EI, 70 eV): *m/z* = 144 [M]^+^.

**1-Phenylpiperidine (10c)** [5]

Yellow oil; yield: 34 mg (42%); ^1^H NMR (400 MHz, CDCl_3_, TMS): δ = 1.55–1.59 (m, 2 H), 1.67–1.73 (m, 4 H), 3.13–3.15 (m, 4 H), 6.79–6.83 (m, 1 H), 6.92–6.94 (m, 2 H), 7.22–7.26 (m, 2 H); MS (EI, 70 eV): *m/z* = 161 [M]^+^.

**Diphenylamine (11c)** [8]

Black solid; yield: 52 mg (62%); ^1^H NMR (400 MHz, CDCl_3_, TMS): δ = 5.70 (s, 1 H), 6.93 (m, 2 H), 7.06–7.08 (m, 4 H), 7.25–7.28 (m, 4 H); MS (EI, 70 eV): *m/z* = 169 [M]^+^.

***N*-(4-Chlorophenyl)benzenamine (12c)** [9]

Pale yellow solid; yield: 61 mg (60%); ^1^H NMR (400 MHz, CDCl_3_, TMS): δ = 5.67 (s, 1 H), 6.95–6.70 (m, 3 H), 7.03–7.05 (m, 2 H), 7.19–7.21 (m, 2 H), 7.27–7.29 (m, 2 H); MS (EI, 70 eV): *m/z* = 203 [M]^+^.

***N*-(4-Bromophenyl)benzenamine (13c)** [9]

Pale red solid; yield: 72 mg (58%); ^1^H NMR (400 MHz, CDCl_3_, TMS): δ = 5.67 (s, 1 H), 6.92–6.98 (m, 3 H), 7.04–7.06 (m, 2 H), 7.289–7.30 (m, 2 H), 7.33–7.35 (m, 2 H); MS (EI, 70 eV): *m/z* = 247 [M]^+^.

***N*-[4-(Trifluoromethyl)phenyl]benzenamine (14c)** [10]

Pale yellow solid; yield: 65 mg (55%); ^1^H NMR (400 MHz, CDCl_3_, TMS): δ = 5.80 (s, 1 H), 7.03–7.08 (m, 3 H), 7.13–7.16 (m, 2 H), 7.30–7.35 (m, 2 H), 7.45–7.48 (m, 2 H); MS (EI, 70 eV): *m/z* = 237 [M]^+^.

***N*-Dodecylbenzenamine (15c)** [11]

Pale yellow solid; yield: 82 mg (63%); ^1^H NMR (400 MHz, CDCl_3_, TMS): δ = 0.86–0.90 (m, 3 H), 1.26–1.32 (m, 18 H), 1.57–1.63 (m, 2 H), 3.08–3.11 (m, 2 H), 3.59 (s, 1 H), 6.59–6.61 (m, 2 H), 6.66–6.70 (m, 1 H), 7.15–7.19 (m, 2 H); MS (EI, 70 eV): *m/z* = 261 [M]^+^.

***N*-Phenylacetamide (16c)** [12]

White solid; yield: 27 mg (40%); ^1^H NMR (400 MHz, CDCl_3_, TMS): δ = 2.15 (m, 3 H), 7.08–7.11 (m, 2 H), 7.26–7.3 (m, 2 H), 7.49–7.51 (m, 2 H), 7.78 (s, 1 H); MS: *m/z* = 135 [M]^+^.

***N*-Phenylnicotinamide (17c)** [13]

White solid; yield: 56 mg (56%); ^1^H NMR (400 MHz, CDCl_3_, TMS): δ = 7.17–7.21 (m, 1 H), 7.37–7.41 (m, 2 H), 7.43–7.46 (m, 1 H), 7.64–7.69 (m, 2 H), 8.09 (s, 1 H), 8.21–8.24 (m, 1 H), 8.76 (m, 1 H), 9.10 (s, 1 H); MS (EI, 70 eV): *m/z* = 198 [M]^+^.

***N*-Phenylbenzamide (18c)** [14]

White solid; yield: 40 mg (41%); ^1^H NMR (400 MHz, CDCl_3_, TMS): δ = 7.14–7.17 (m, 1 H), 7.37–7.39 (m, 2 H), 7.48–7.51 (m, 2 H), 7.55–7.57 (m, 1 H), 7.64–7.65 (m, 2 H), 7.82 (s, 1 H), 7.87–7.88 (m, H); MS (EI, 70 eV): *m/z* = 197 [M]^+^.

**4-Methyl-*N*-phenylbenzenesulfonamide (19c)** [15]

White solid; yield: 37 mg (30%); ^1^H NMR (400 MHz, CDCl_3_, TMS): δ = 2.37 (m, 3 H), 6.68 (s, 1 H), 7.05–7.13 (m, 3 H), 7.21–7.26 (m, 4 H), 7.64–7.66 (m, 2 H); MS (EI, 70 eV): *m/z* = 247 [M]^+^.

**References:**

1. Yadav, L. D. S.; Yadav, B. S.; Rai, V. K. *Synthesis* **2006,** 1868–1872. doi:[10.1055/s-2006-942369](http://dx.doi.org/10.1055/s-2006-942369)
2. Heravi, M. M.; Shoar, R. H.; Pedram, L. *J. Mol. Catal. A: Chem.* **2005,** *231,* 89–91. doi:[10.1016/j.molcata.2005.01.005](http://dx.doi.org/10.1016/j.molcata.2005.01.005)
3. Koltunov, K. Yu.; Prakash, G. K. S.; Rasul, G.; Olah, G. A. *Eur. J. Org. Chem.* **2006,** 4861–4866. doi:[10.1002/ejoc.200600486](http://dx.doi.org/10.1002/ejoc.200600486)
4. Maheswaran, H.; Krishna, G. G.; Prasanth, K. L.; Srinivas, V.; Chaitanya, G. K.; Bhanuprakash, K. *Tetrahedron* **2008,** *64,* 2471–2479. doi:[10.1016/j.tet.2007.12.050](http://dx.doi.org/10.1016/j.tet.2007.12.050)
5. Yang, M.; Liu, F. *J. Org. Chem.* **2007,** *72,* 8969–8971. doi:[10.1021/jo0712291](http://dx.doi.org/10.1021/jo0712291)
6. Chen, W.; Zhang, Y.; Zhu, L.; Lan, J.; Xie, R.; You, J. *J. Am. Chem. Soc.* **2007,** *129,* 13879–13886. doi:[10.1021/ja073633n](http://dx.doi.org/10.1021/ja073633n)
7. Elliott, G. I.; Konopelski, J. P. *Org. Lett.* **2000,** *2,* 3055–3057. doi:[10.1021/ol006271w](http://dx.doi.org/10.1021/ol006271w)
8. Gao, C.-J.; Yang, L.-M. *J. Org. Chem.* **2008,** *73,* 1624–1627. doi:[10.1021/jo7022558](http://dx.doi.org/10.1021/jo7022558)
9. Carroll, M. A.; Wood, R. A. *Tetrahedron* **2007,** *63,* 11349–11354. doi:[10.1016/j.tet.2007.08.076](http://dx.doi.org/10.1016/j.tet.2007.08.076)
10. Hagopian, L. E.; Campbell, A. N.; Golen, J. A.; Rheingold, A. L.; Nataro, C. *J. Organomet. Chem.* **2006,** *691,* 4890–4900. doi:[10.1016/j.jorganchem.2006.08.030](http://dx.doi.org/10.1016/j.jorganchem.2006.08.030)
11. Jiang, D.; Fu, H.; Jiang, Y.; Zhao, Y. *J. Org. Chem.* **2007,** *72,* 672–674. doi:[10.1021/jo062060e](http://dx.doi.org/10.1021/jo062060e)
12. Thakuria, H.; Borah, B. M.; Das, G. *J. Mol. Catal. A: Chem.* **2007,** *274,* 1–10. doi:[10.1016/j.molcata.2007.04.024](http://dx.doi.org/10.1016/j.molcata.2007.04.024)
13. Lv, X.; Bao, W. *J. Org. Chem.* **2007,** *72,* 3863–3867. doi:[10.1021/jo070443m](http://dx.doi.org/10.1021/jo070443m)
14. Correa, A.; Bolm, C. *Angew. Chem., Int. Ed.* **2007,** *46,* 8862–8865. doi:[10.1002/anie.200703299](http://dx.doi.org/10.1002/anie.200703299)
15. Purushottamachar, P.; Khandelwal, A.; Vasaitis, T. S.; Bruno, R. D.; Gediya, L. K.; Njar, V. C. O. *Bioorg. Med. Chem.* **2008,** *16,* 3519–3529. doi:[10.1016/j.bmc.2008.02.031](http://dx.doi.org/10.1016/j.bmc.2008.02.031)

**Copy of H NMR spectra for the products and starting materials**


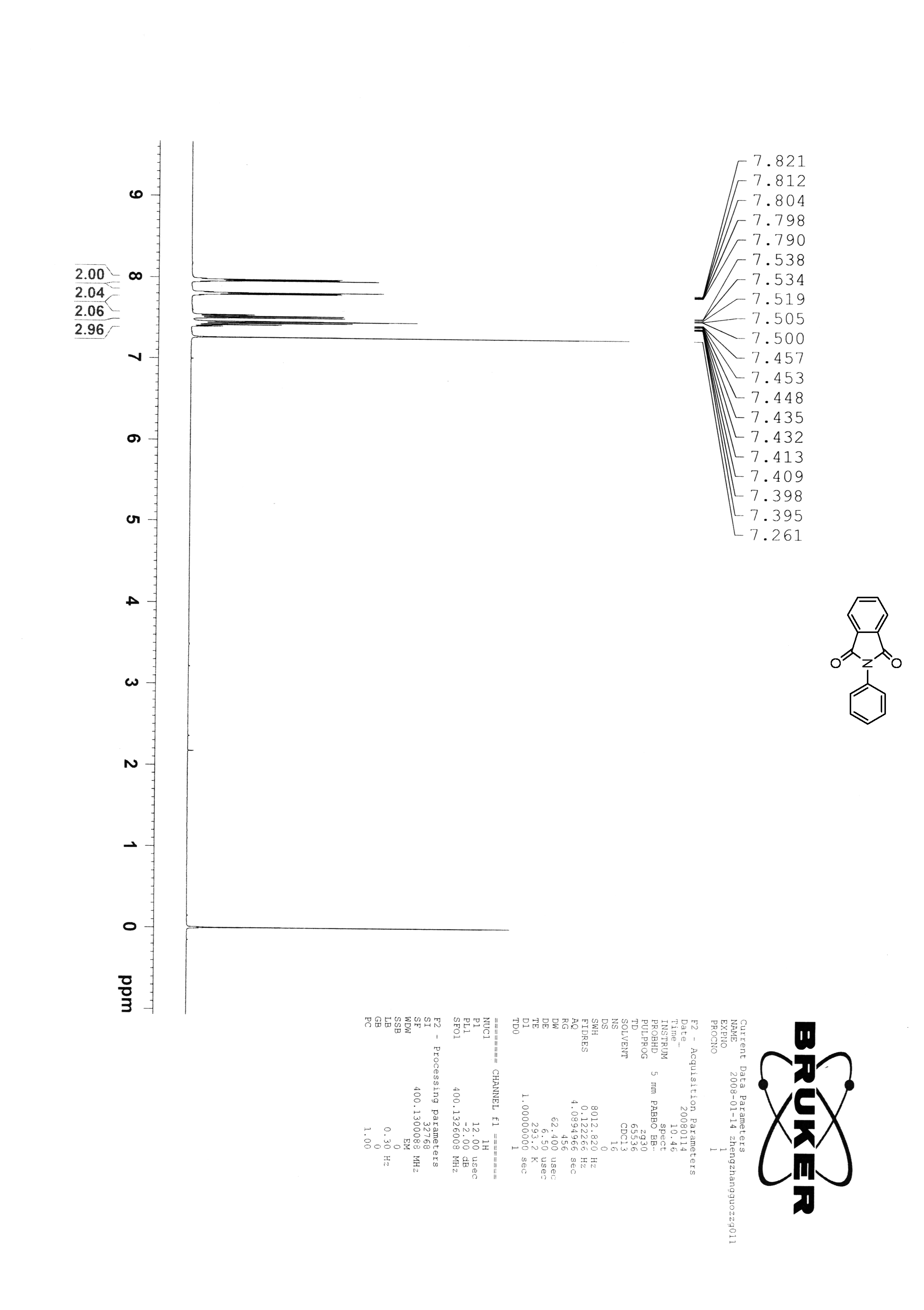
Figure S1 ^1^H NMR of compound **1c**


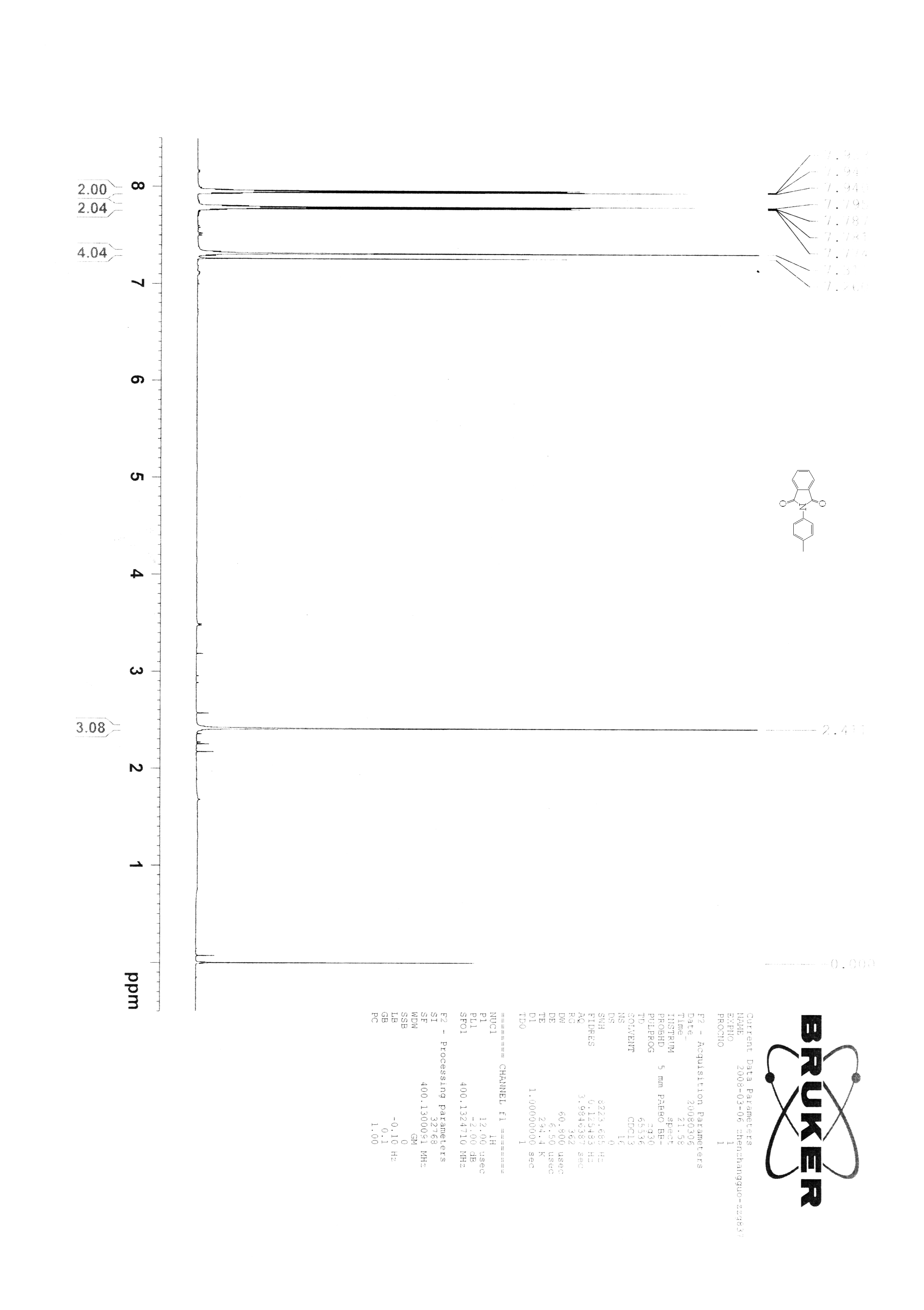
Figure S2 ^1^H NMR of compound **2c**


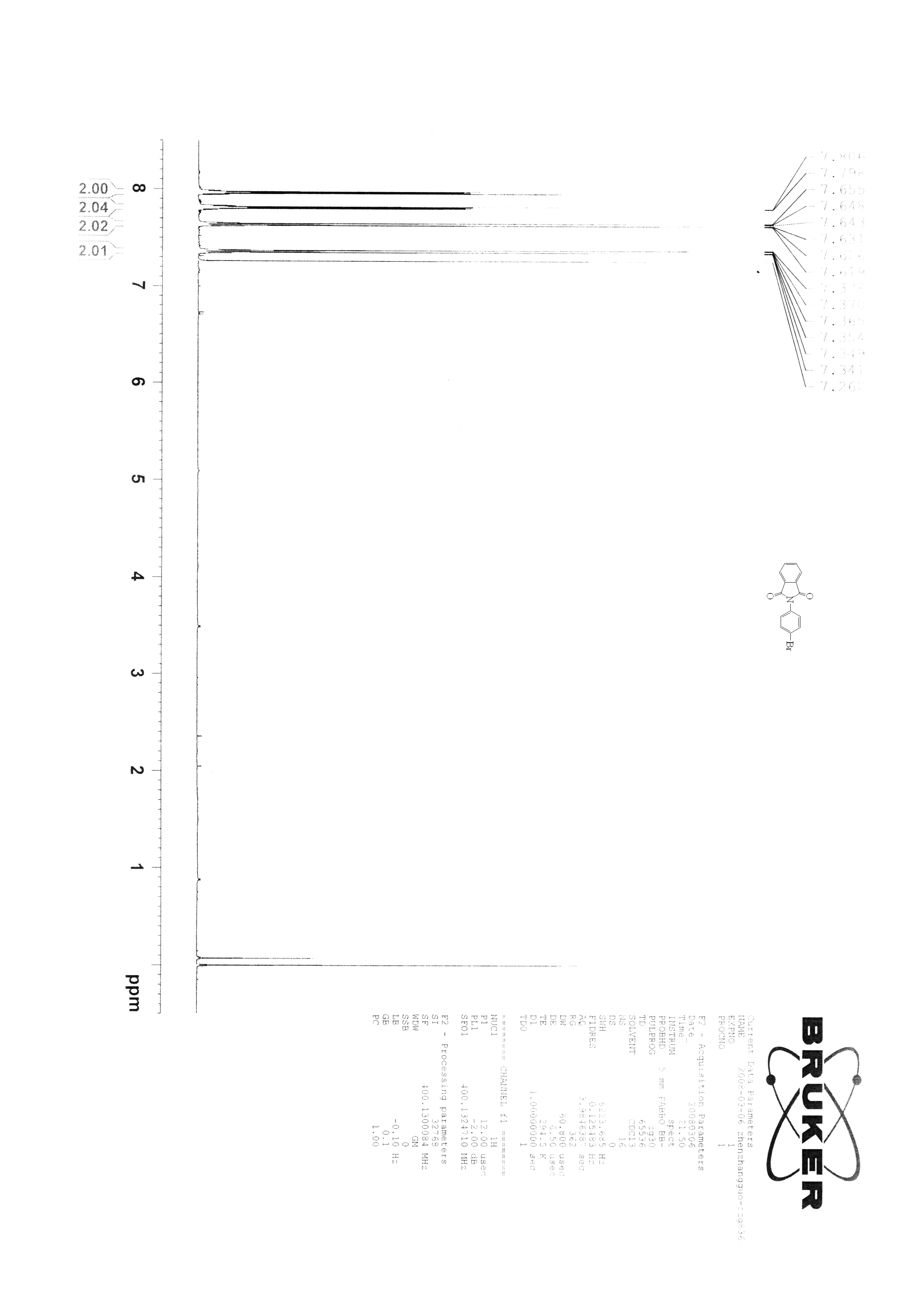
Figure S3 ^1^H NMR of compound **3c**


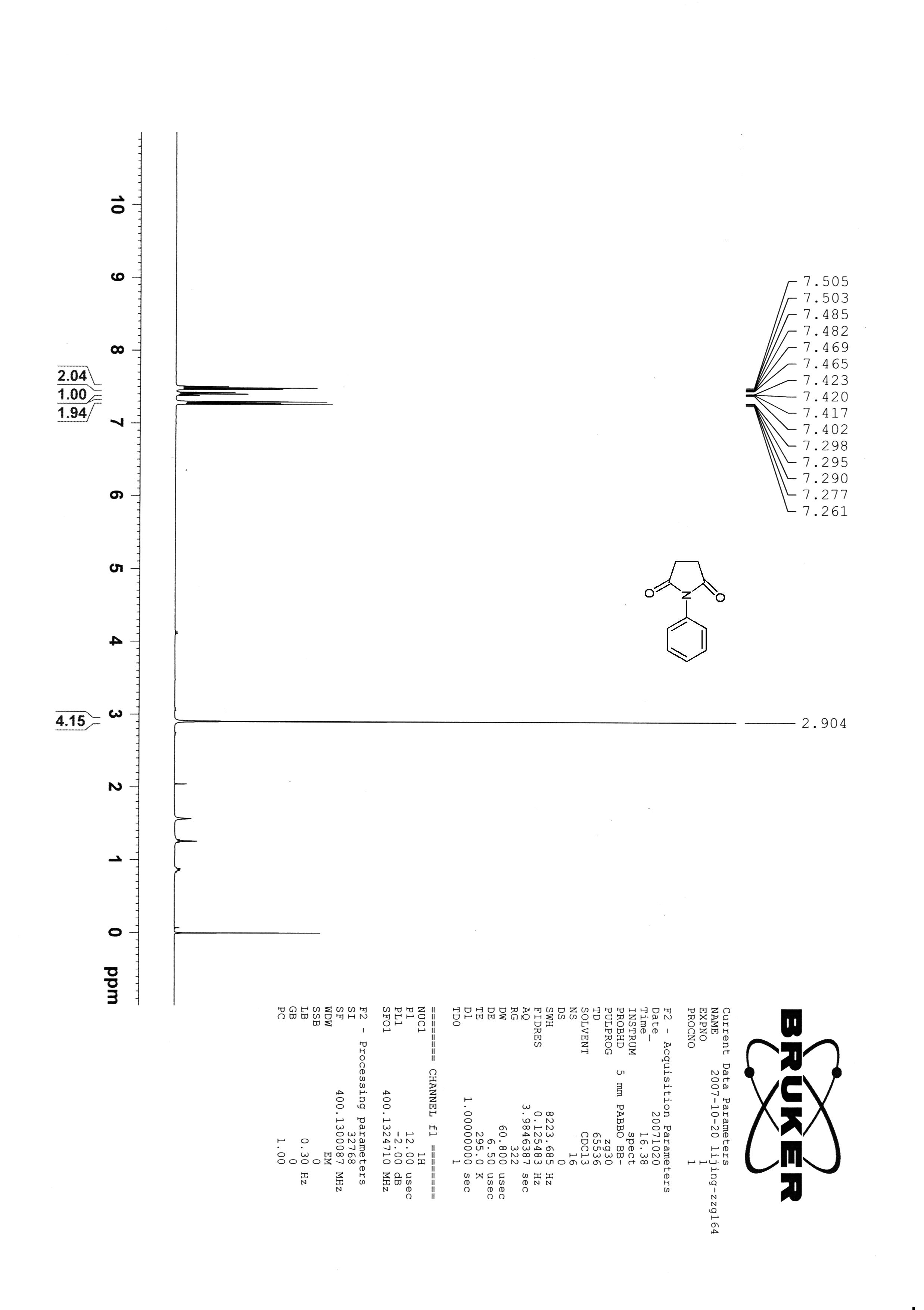


Figure S4 ^1^H NMR of compound **4c**


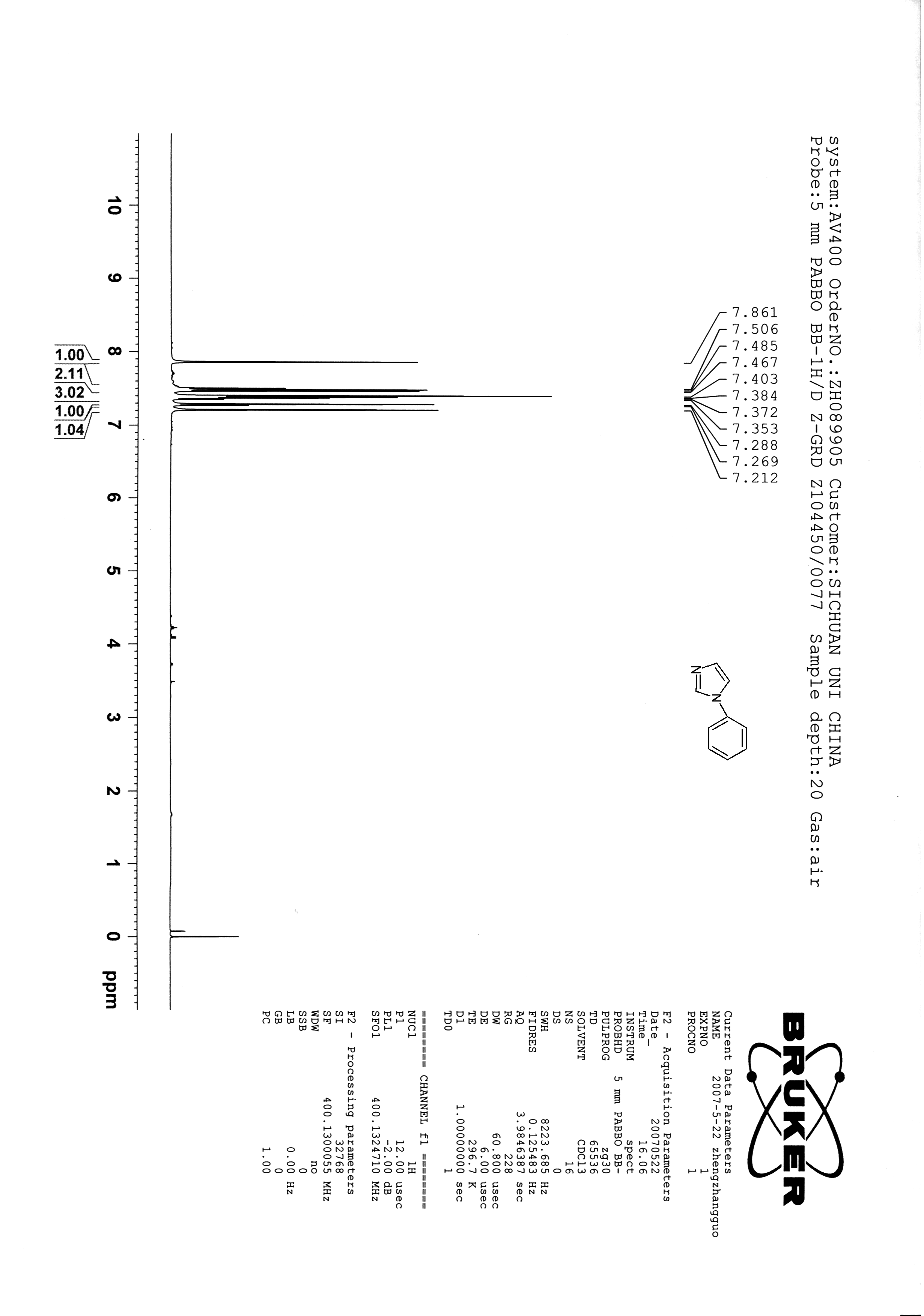


Figure S5 ^1^H NMR of compound **5c**


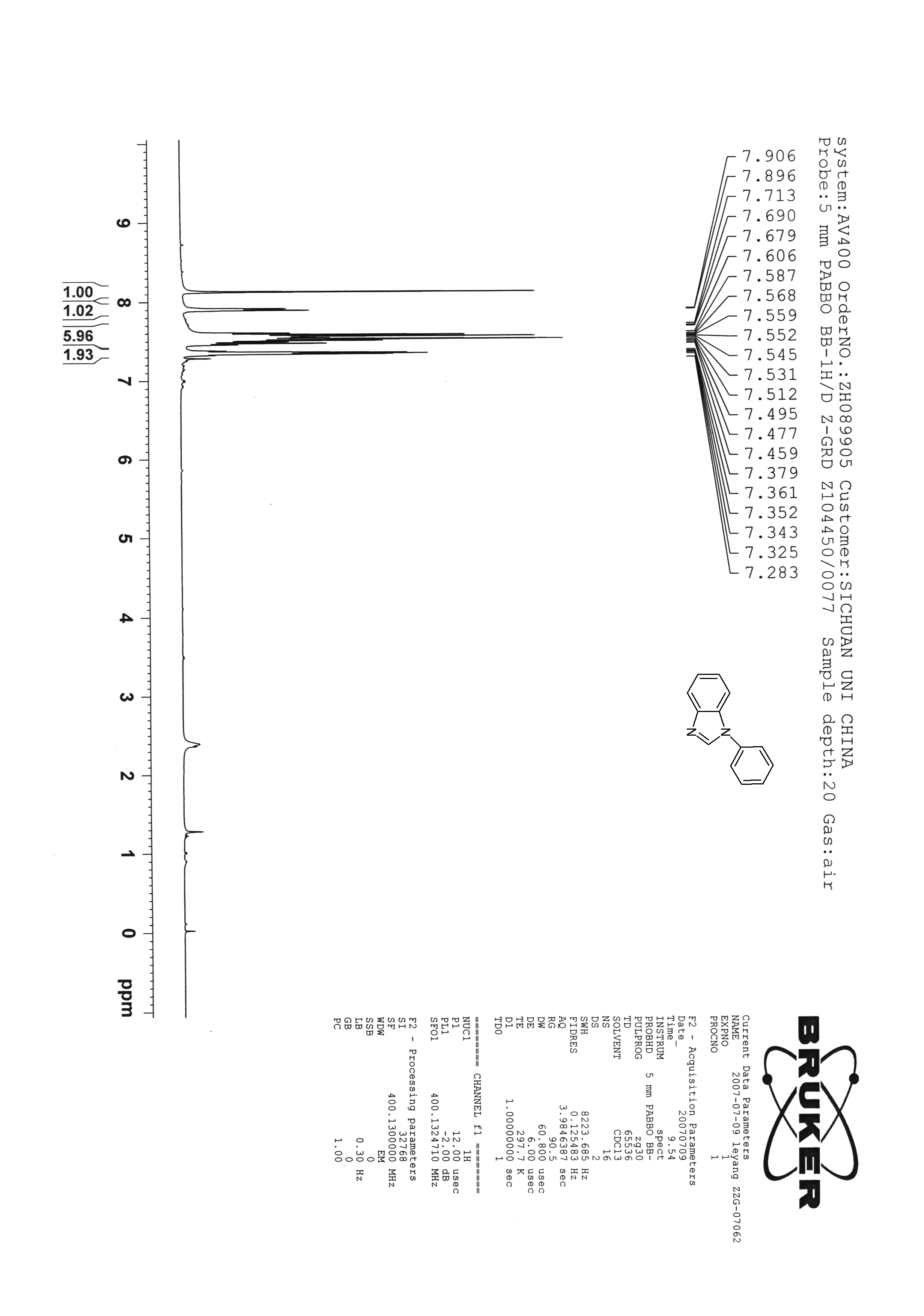


Figure S6 ^1^H NMR of compound **6c**


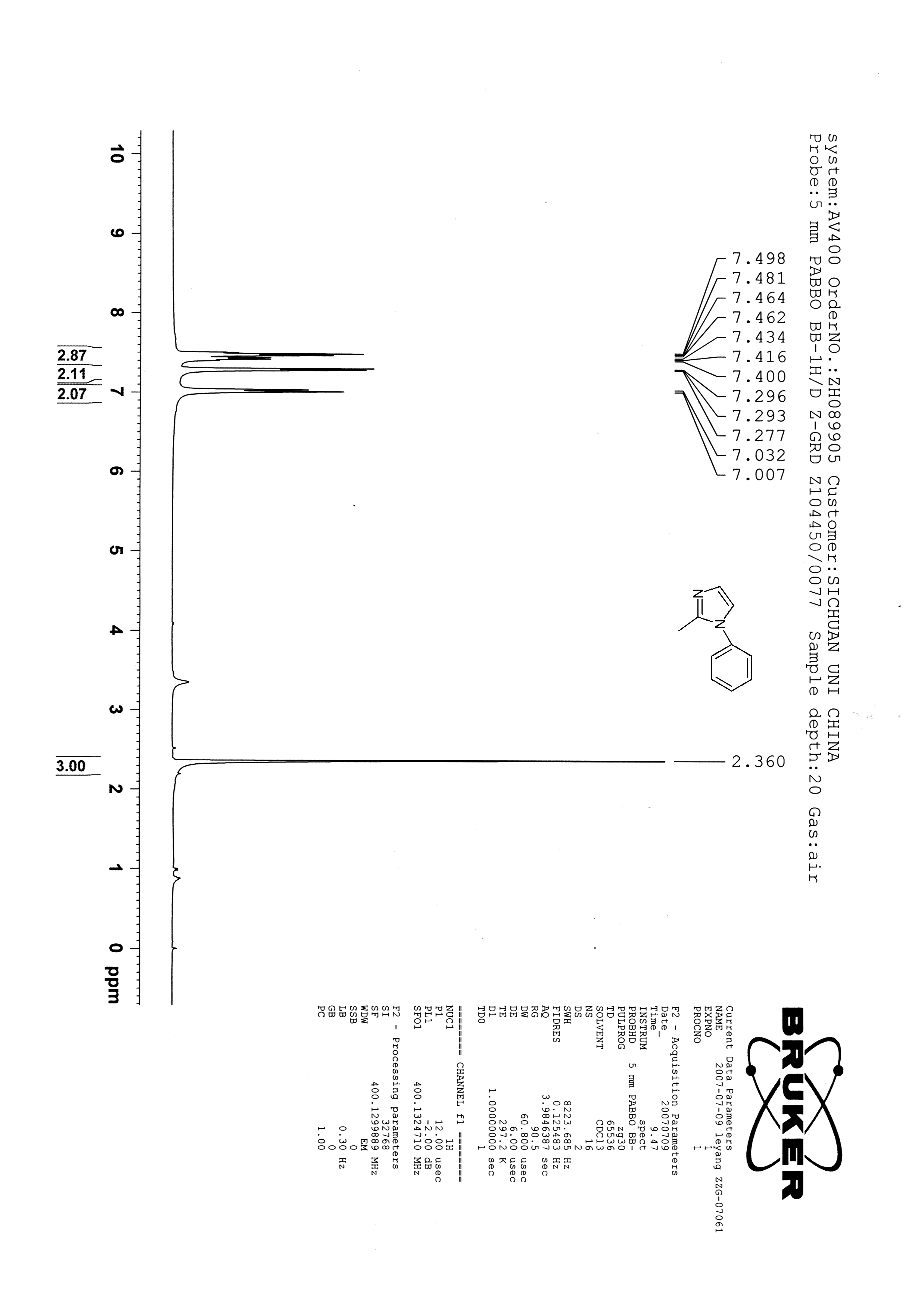


Figure S7 ^1^H NMR of compound **7c**


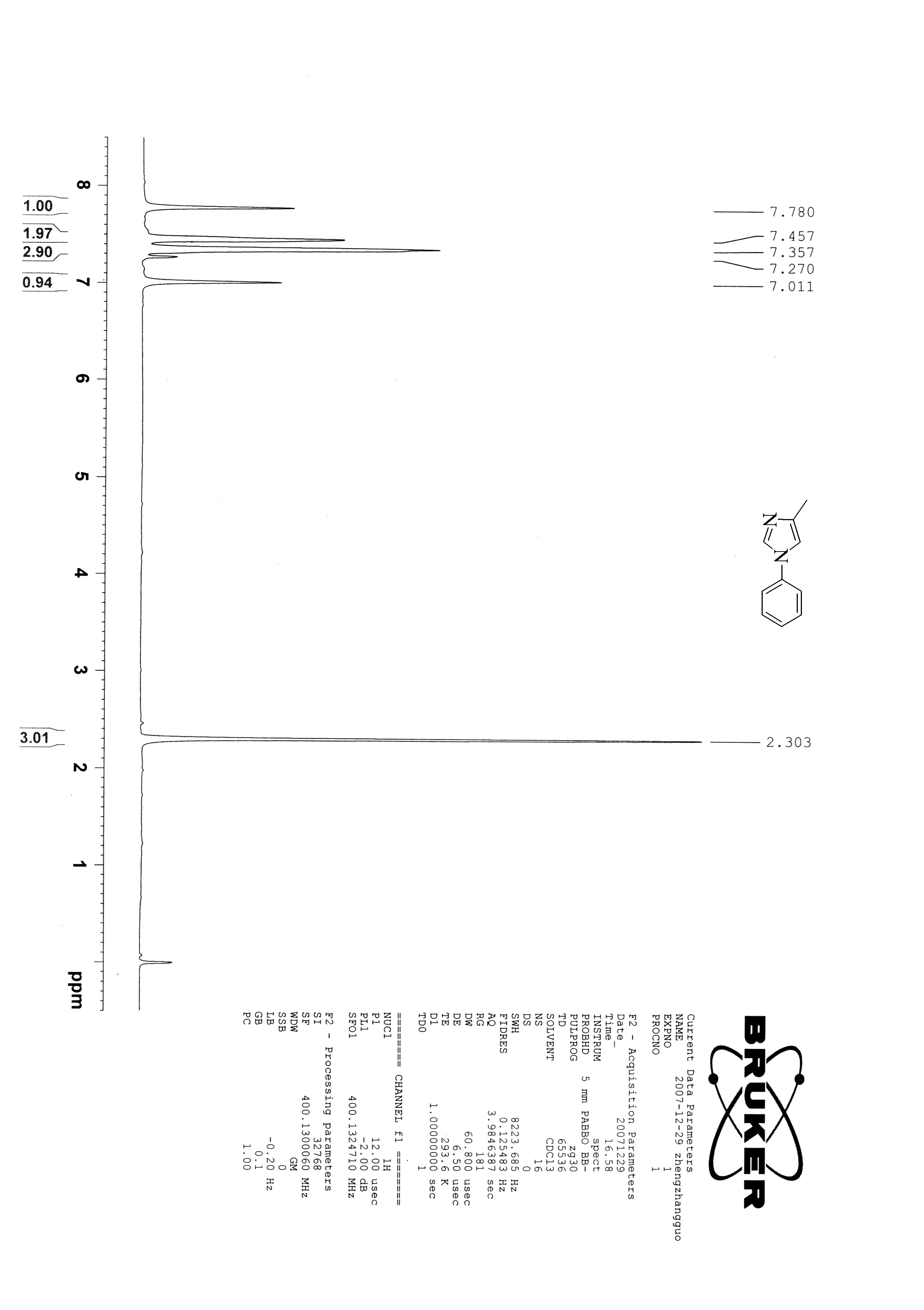


Figure S8 ^1^H NMR of compound **8c**


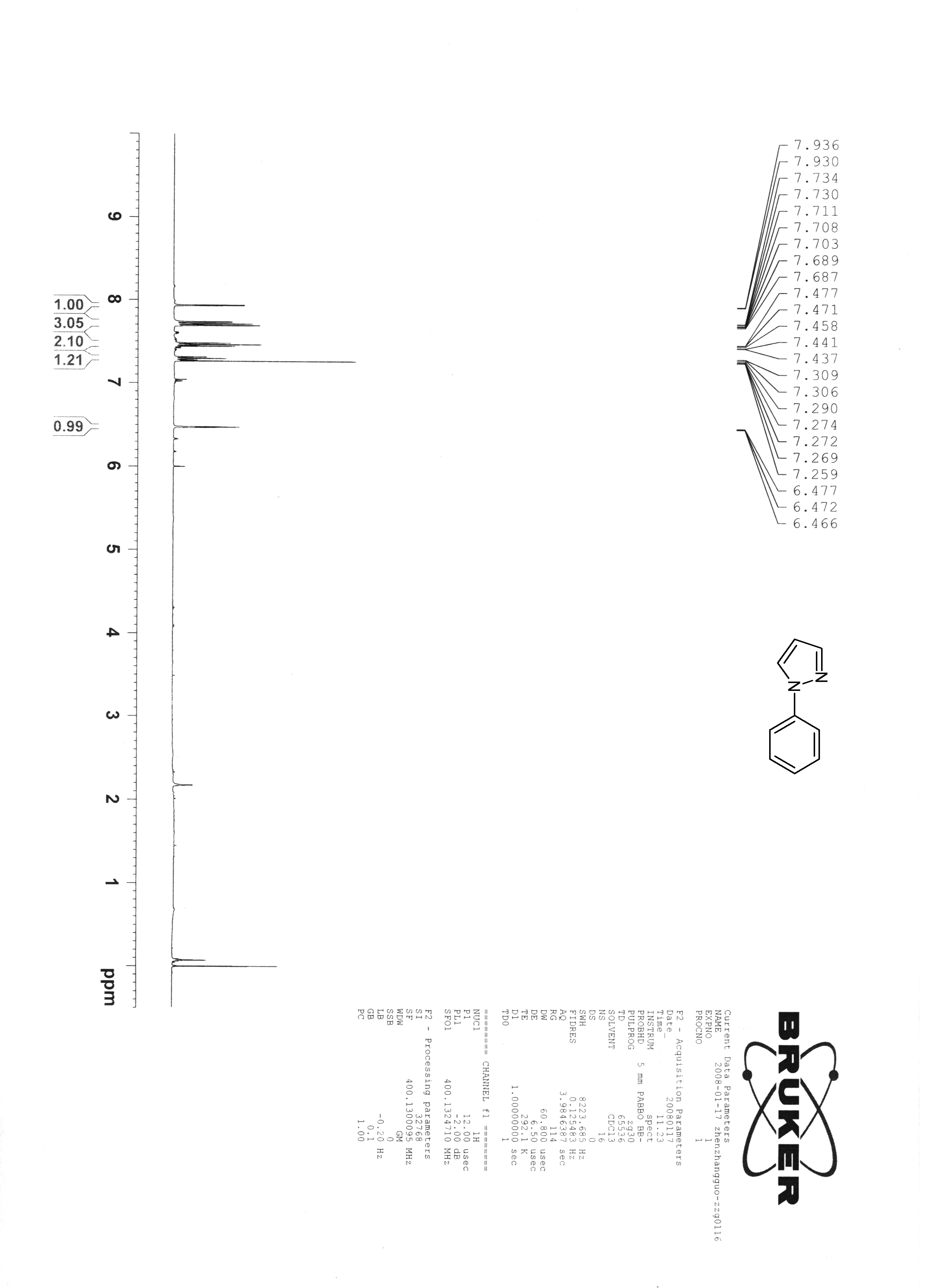


Figure S9 ^1^H NMR of compound **9c**


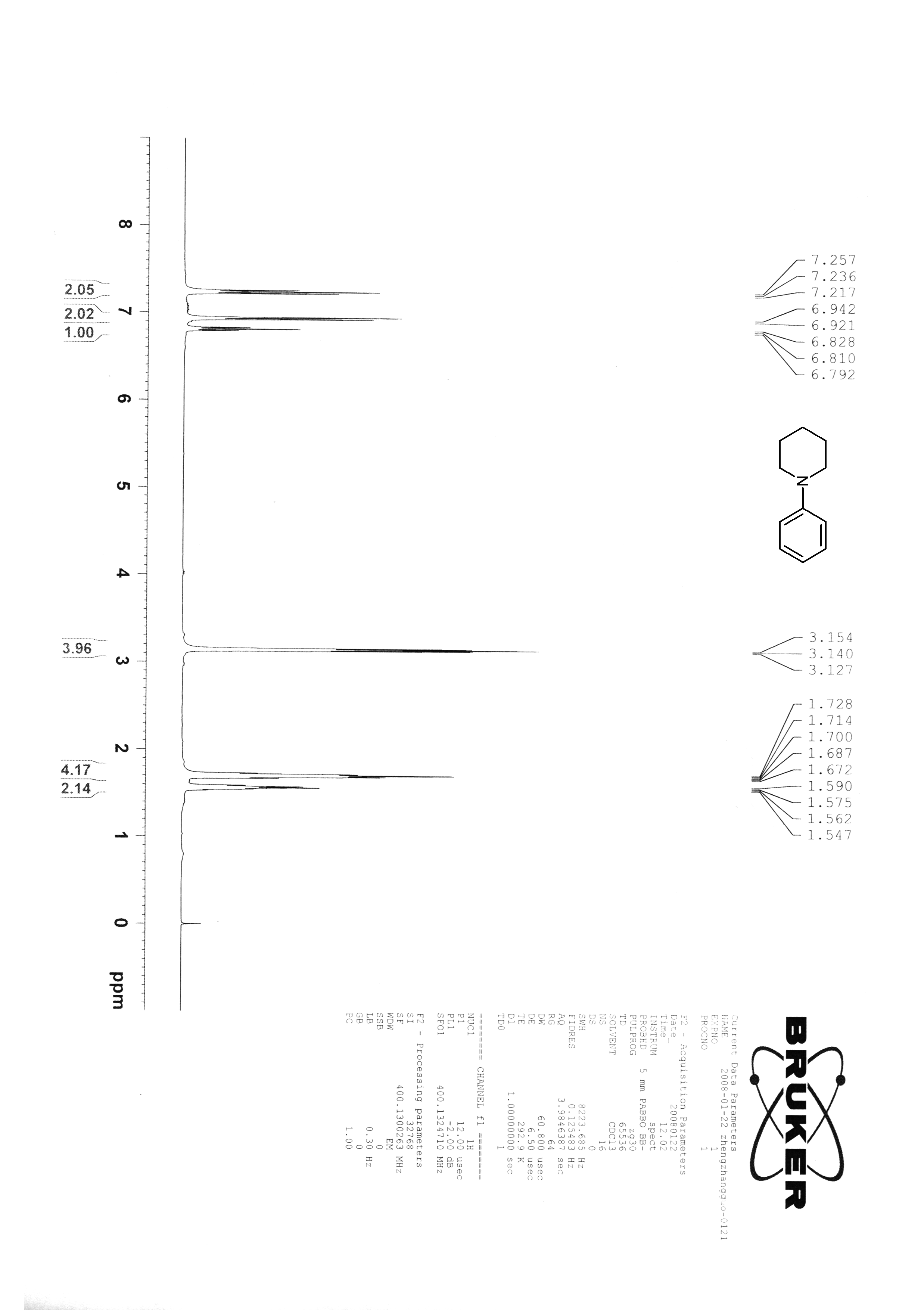


Figure S10 ^1^H NMR of compound **10c**


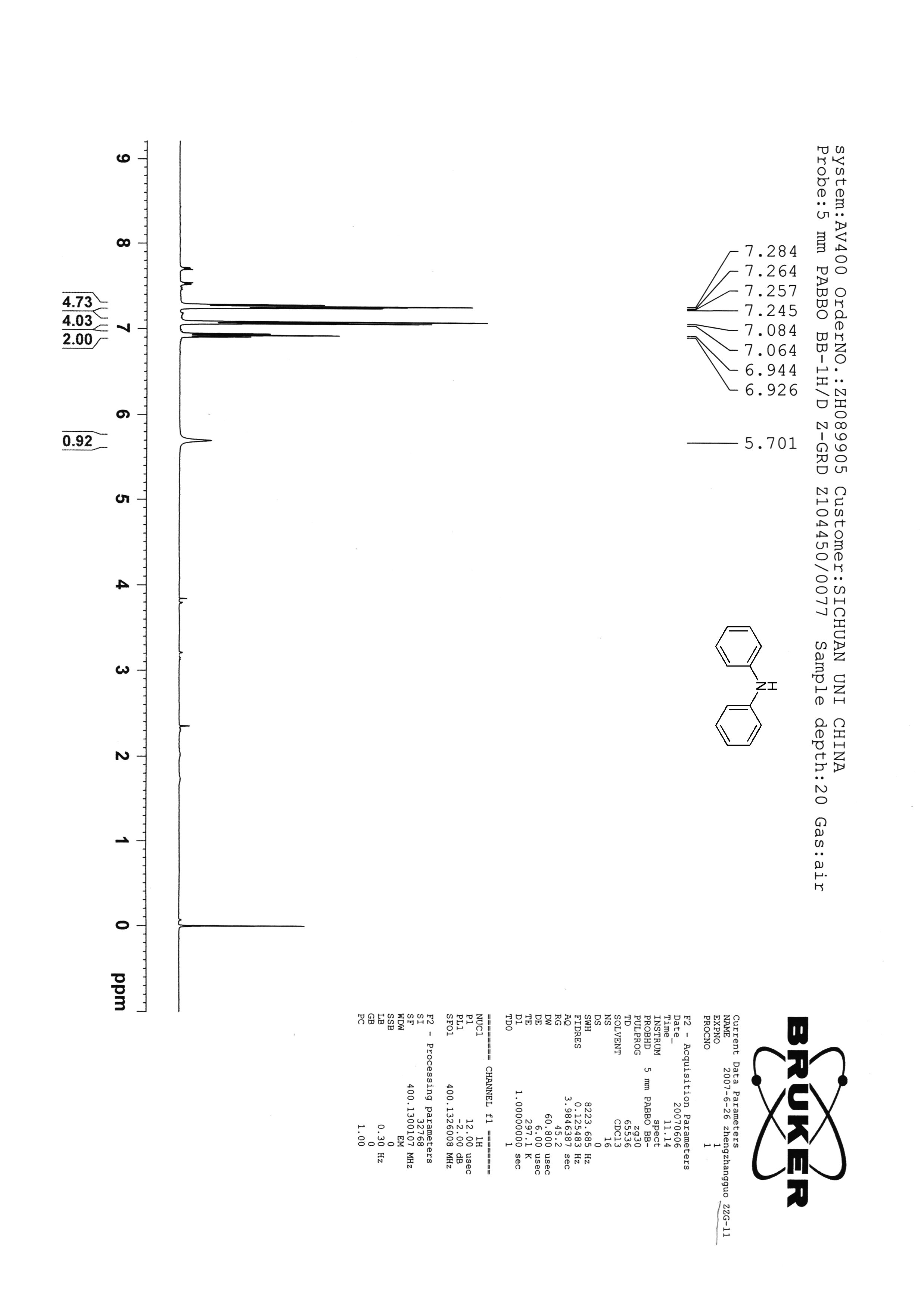


Figure S11 ^1^H NMR of compound **11c**


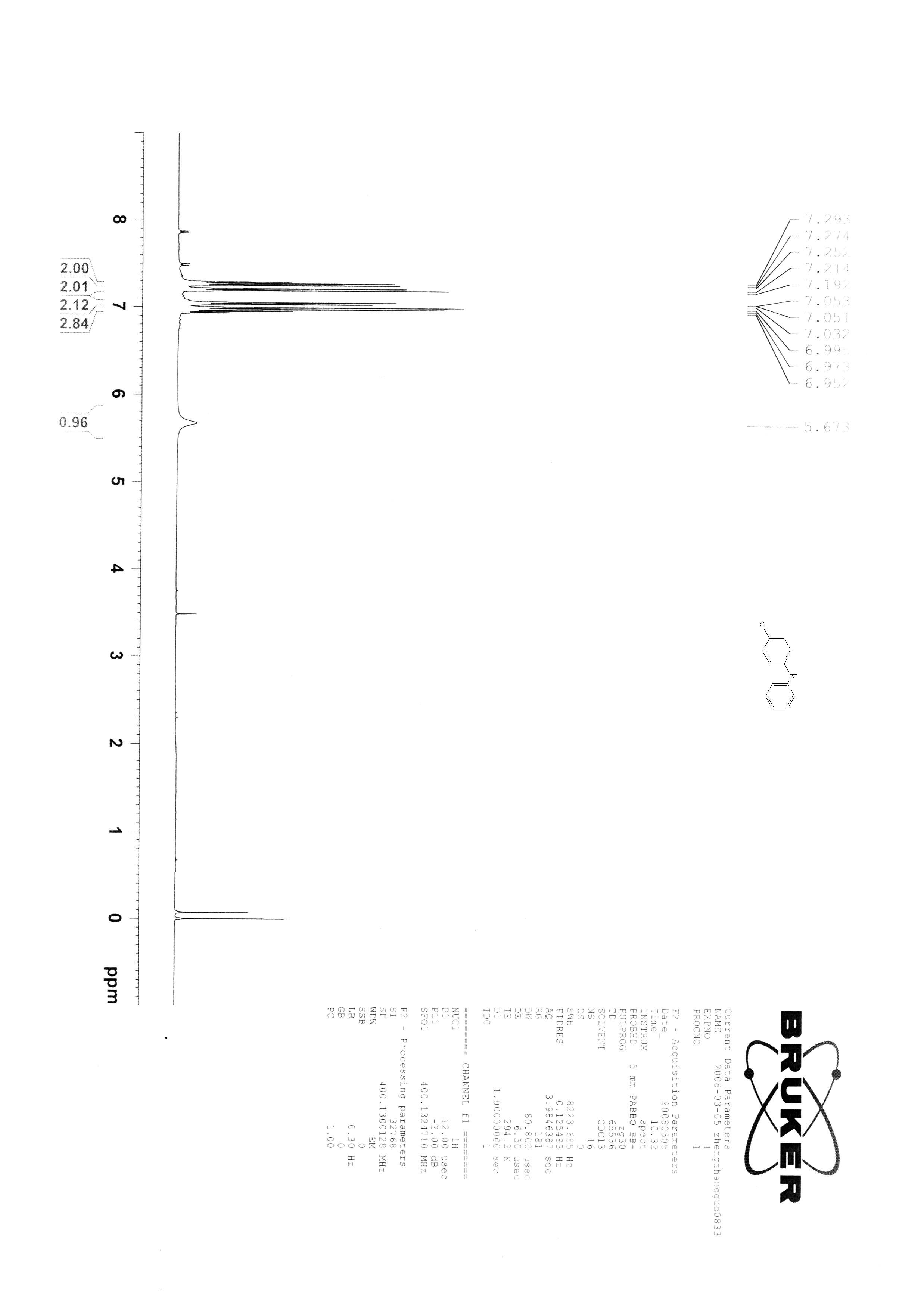


Figure S12 ^1^H NMR of compound **12c**


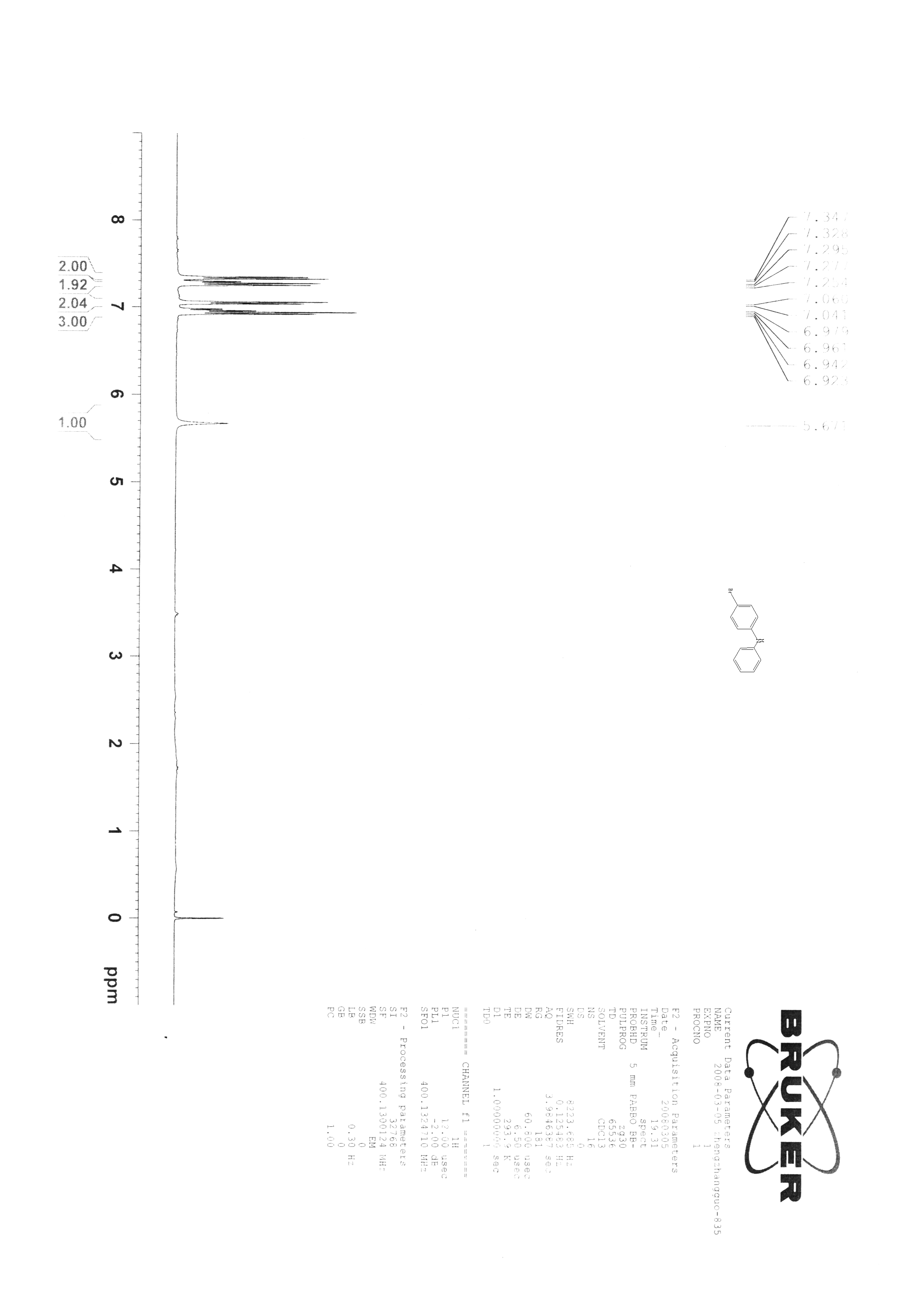


Figure S13 ^1^H NMR of compound **13c**


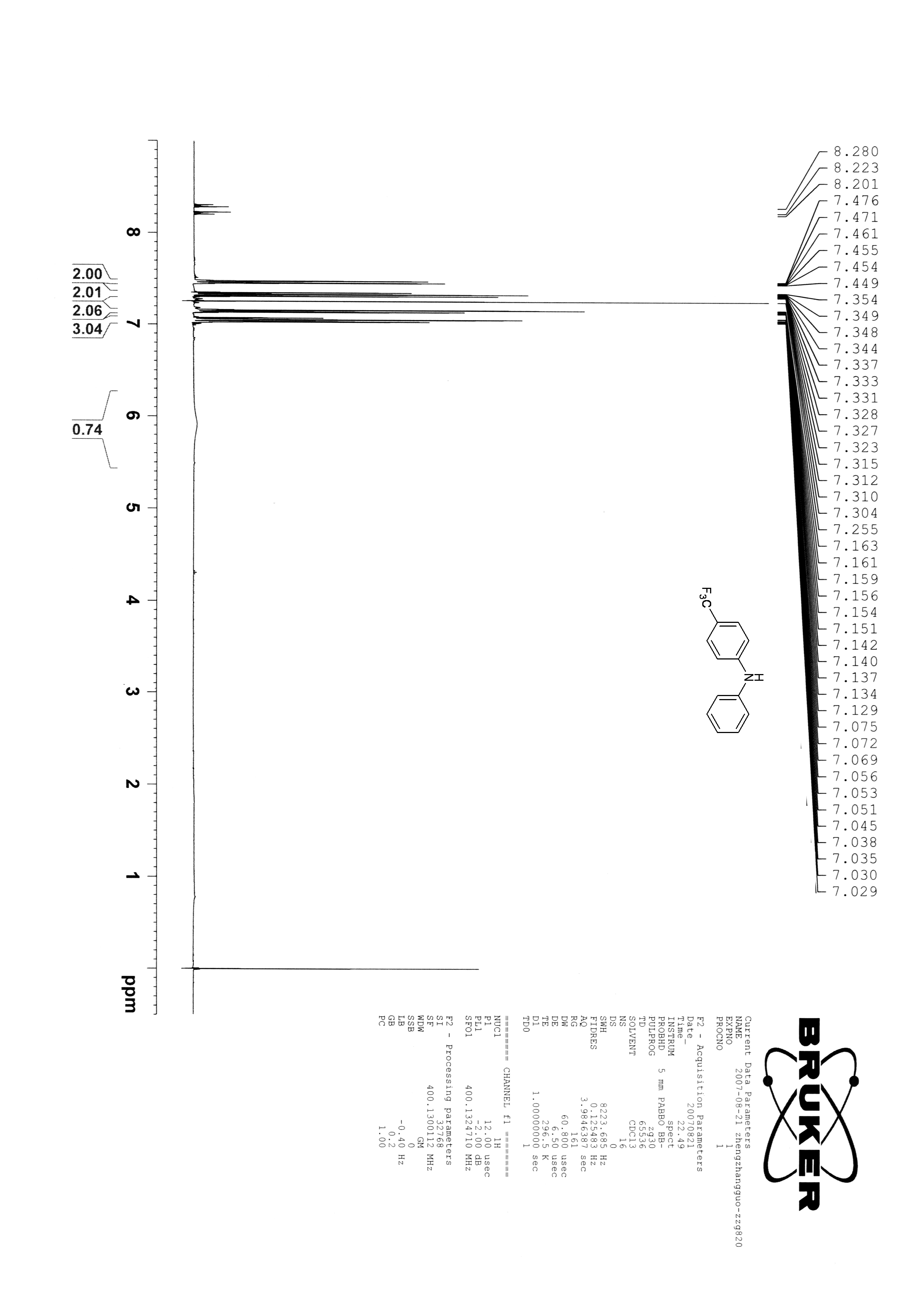


Figure S14 ^1^H NMR of compound **14c**


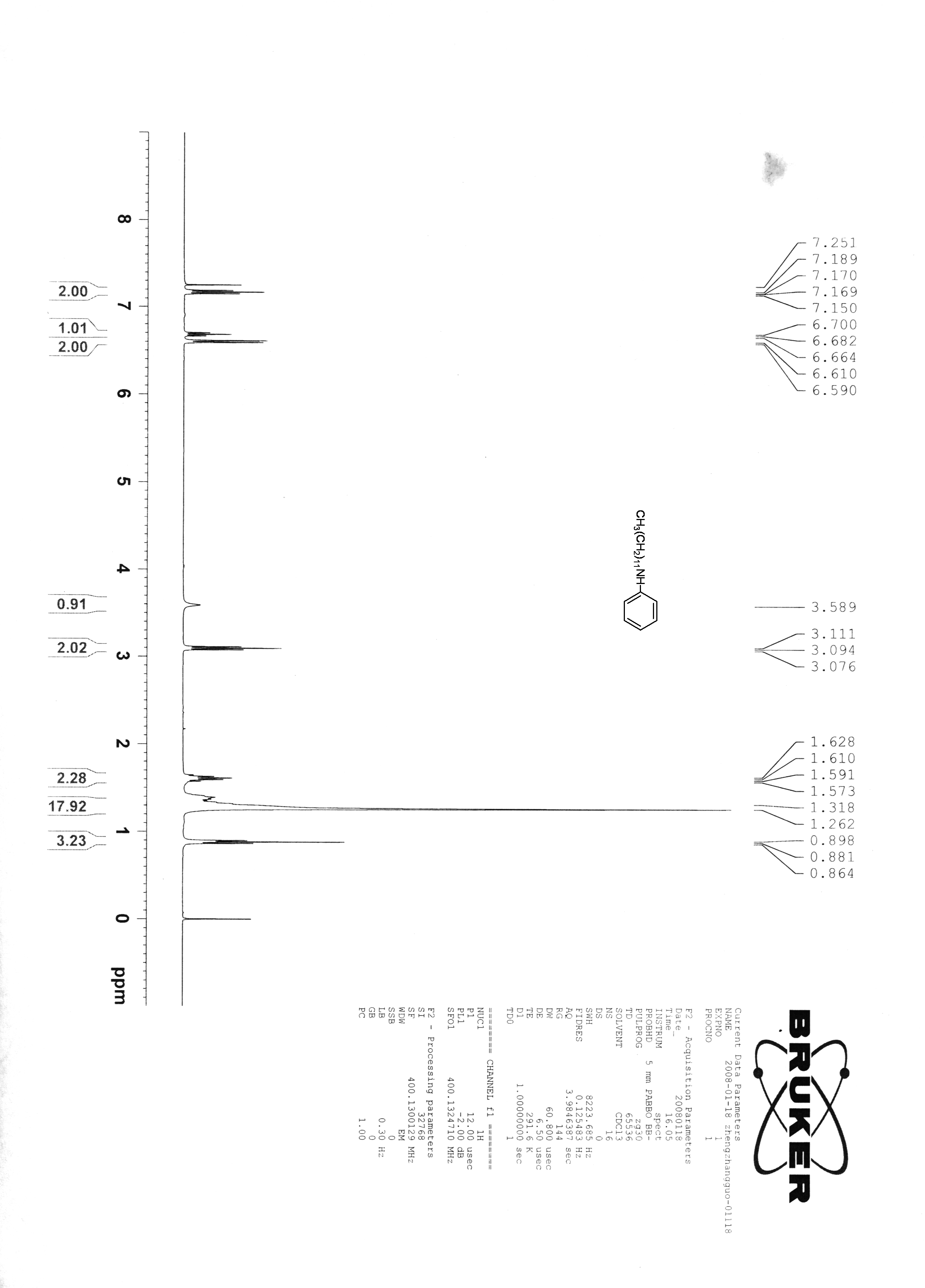


Figure S15 ^1^H NMR of compound **15c**


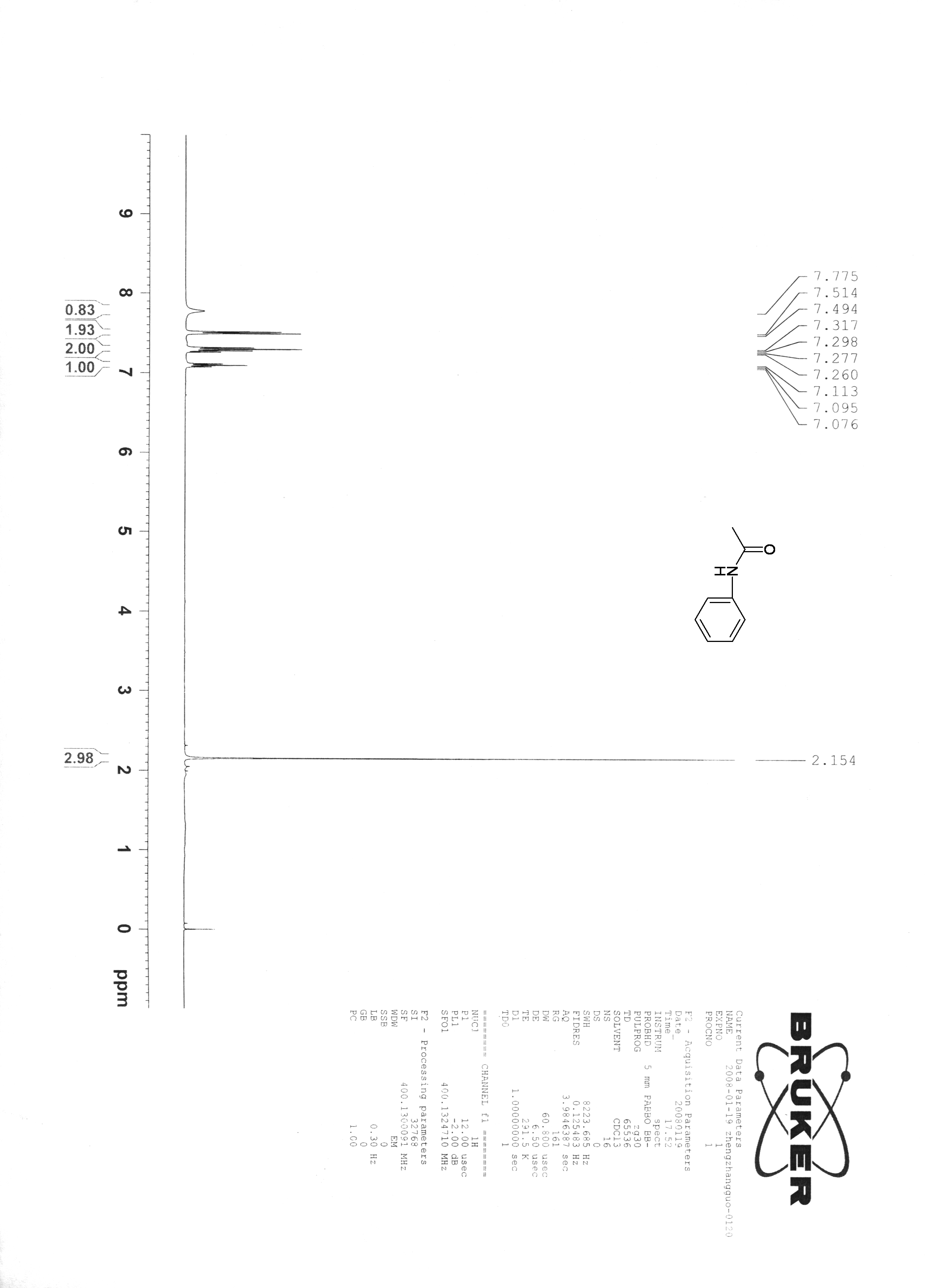


Figure S16 ^1^H NMR of compound **16c**


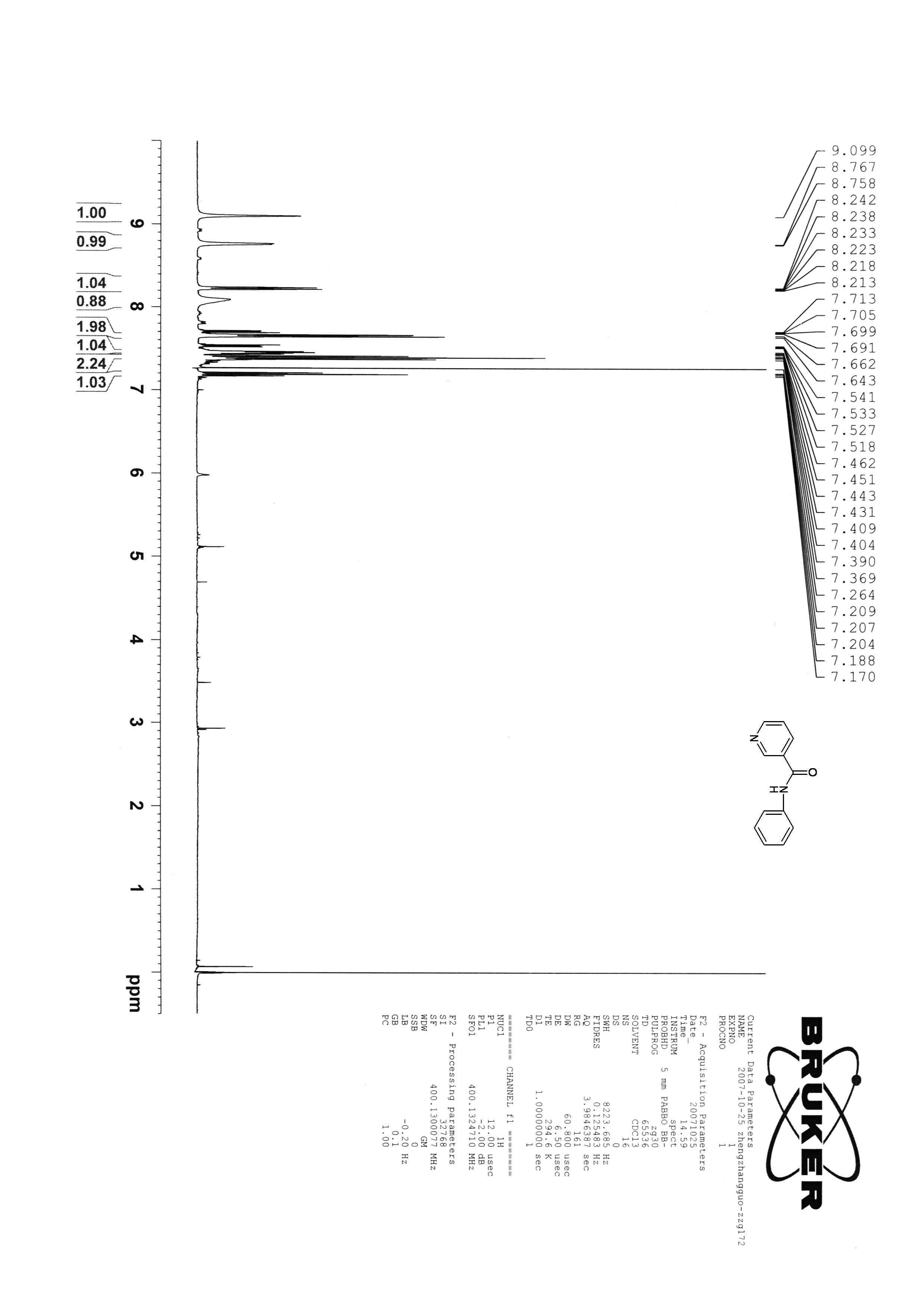


Figure S17 ^1^H NMR of compound **17c**


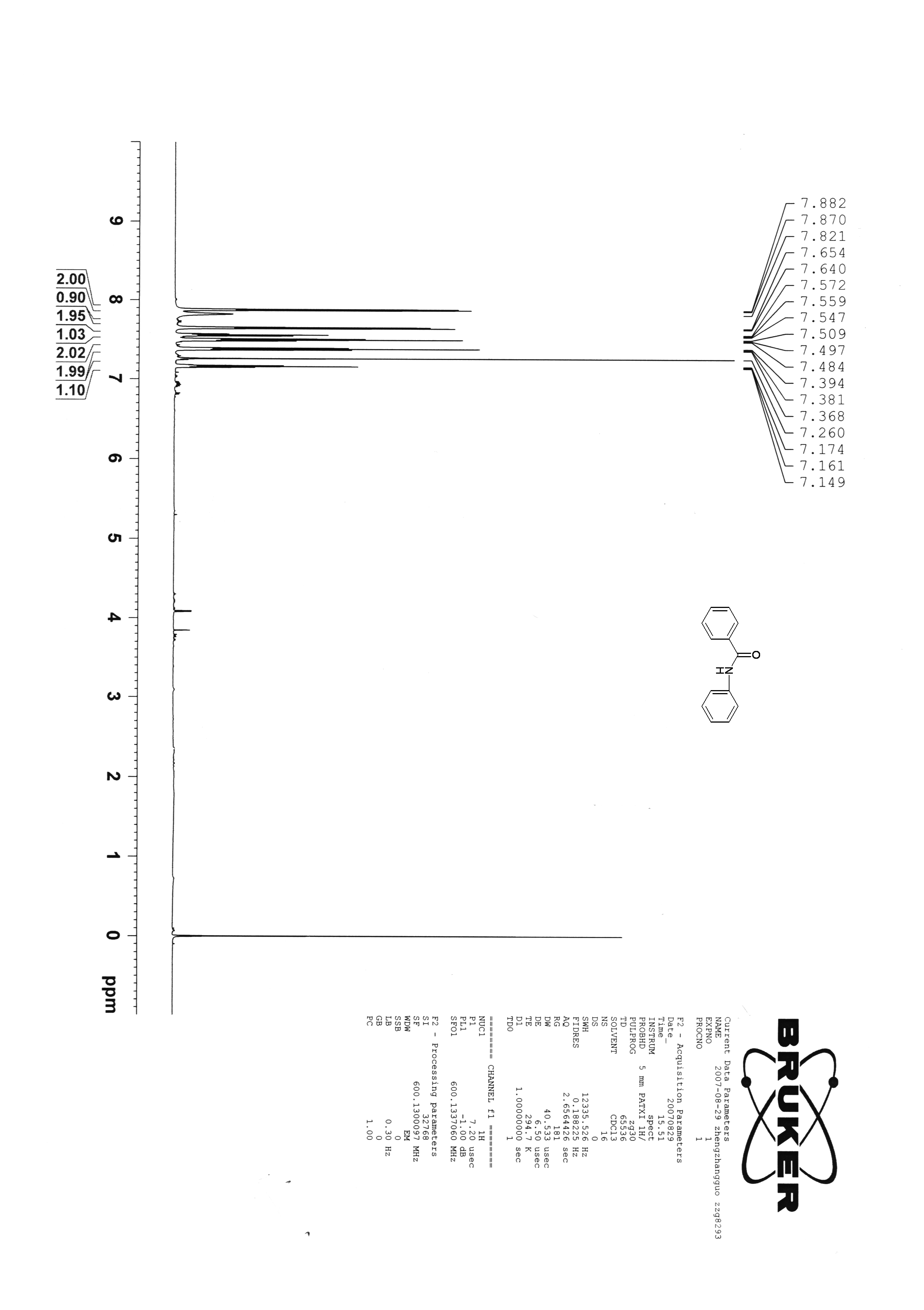


Figure S18 ^1^H NMR of compound **18c**


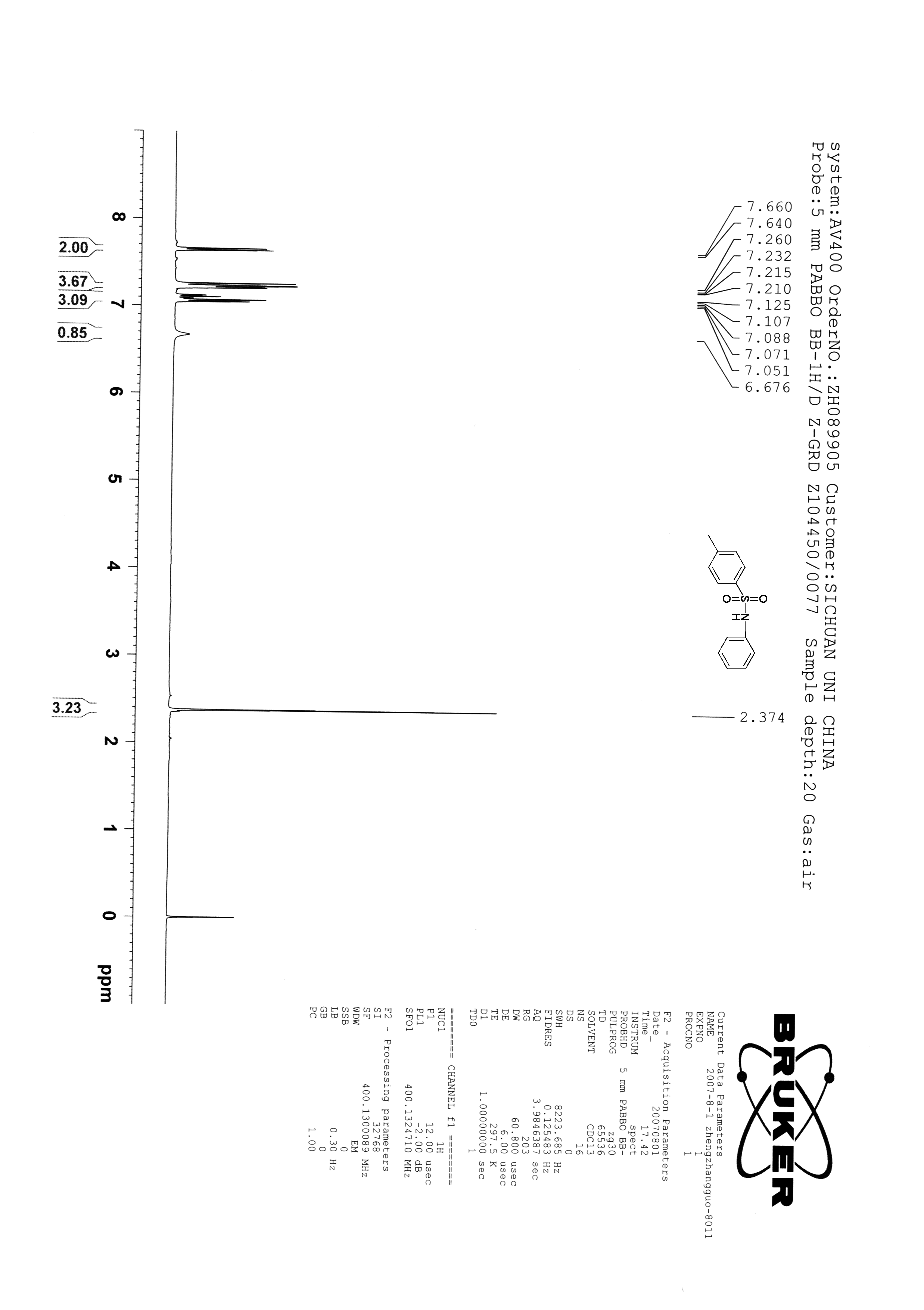


Figure S19 ^1^H NMR of compound **19c**


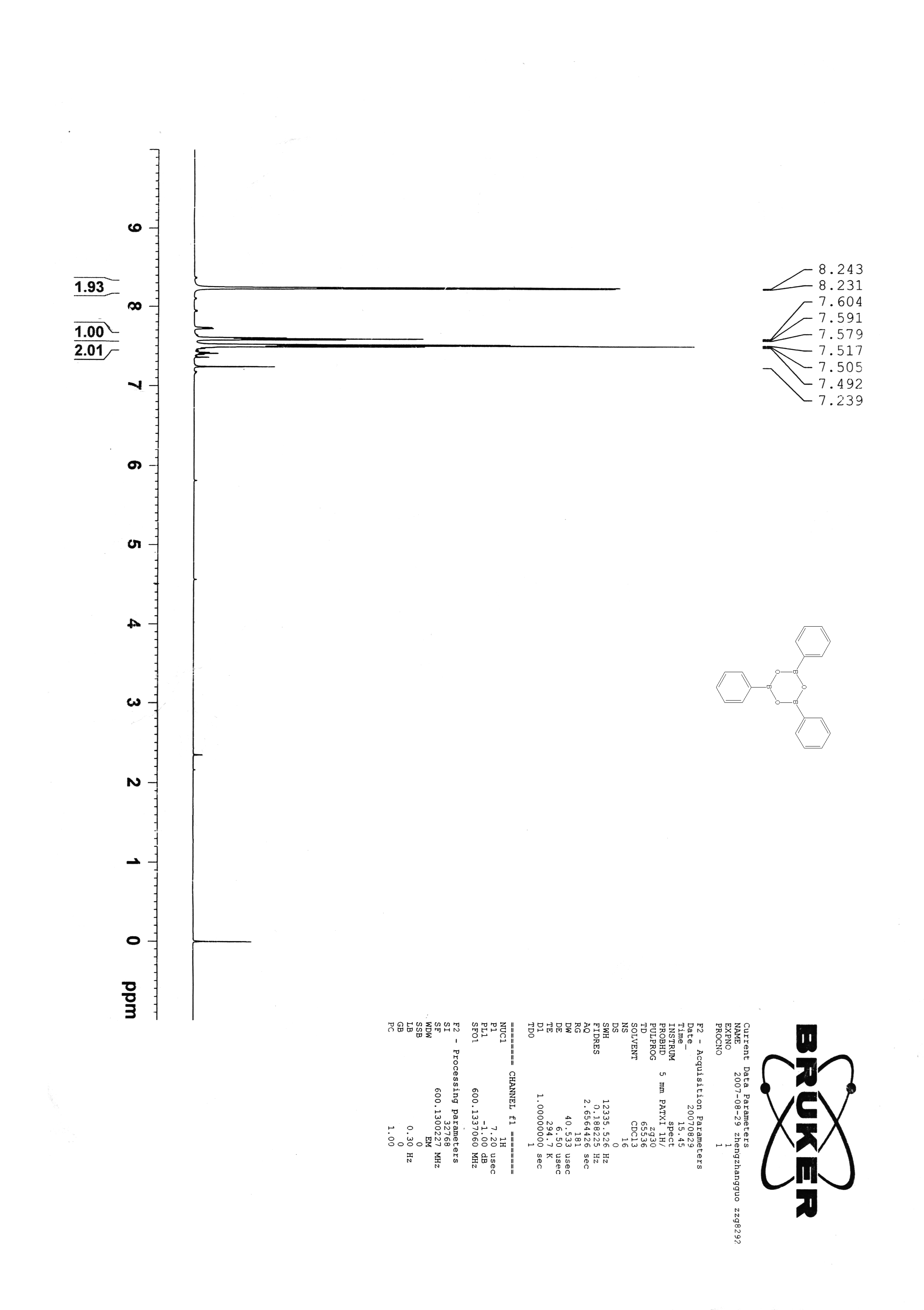


Figure S20 ^1^H NMR of compound **1b**


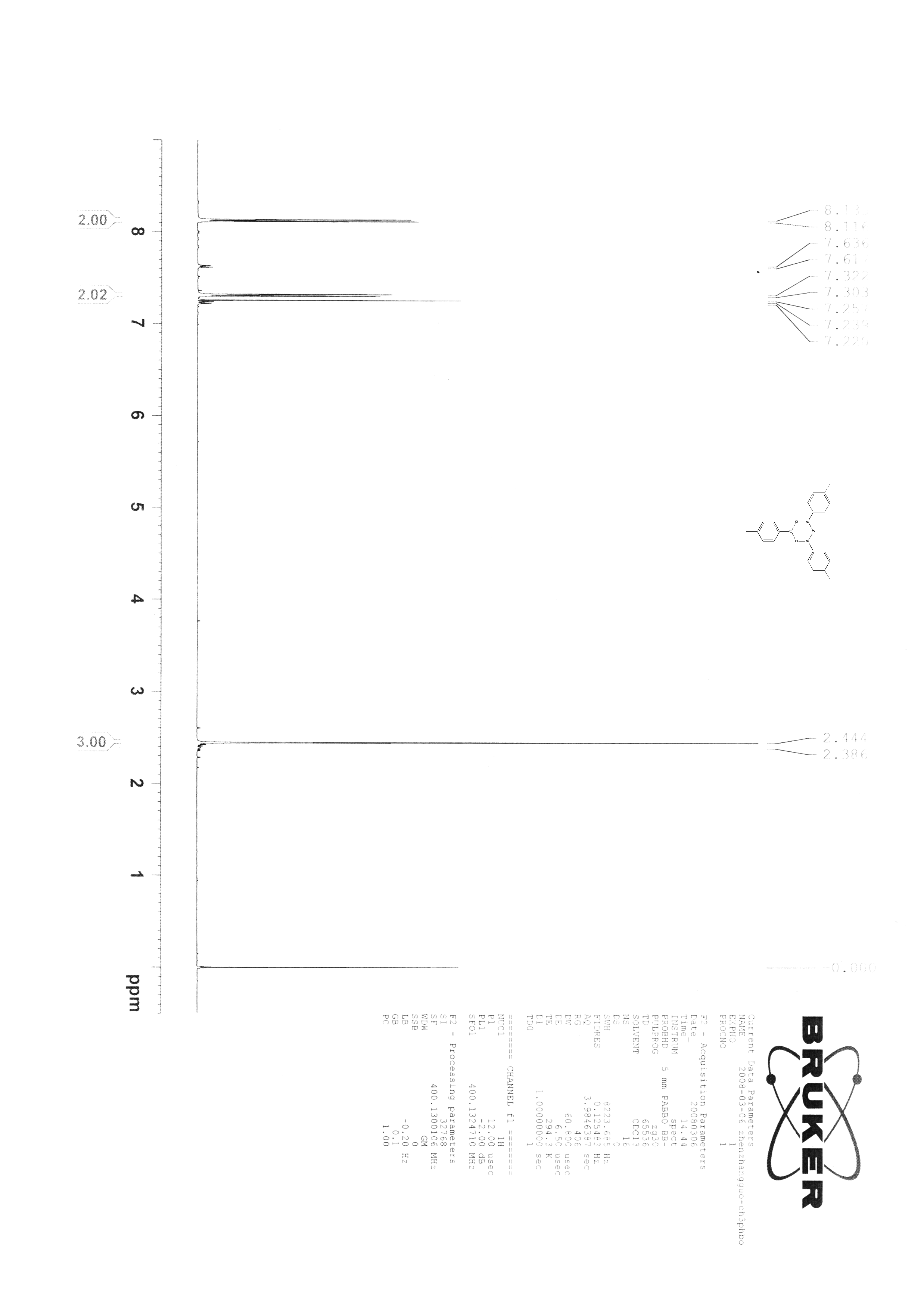


Figure S21 ^1^H NMR of compound **2b**


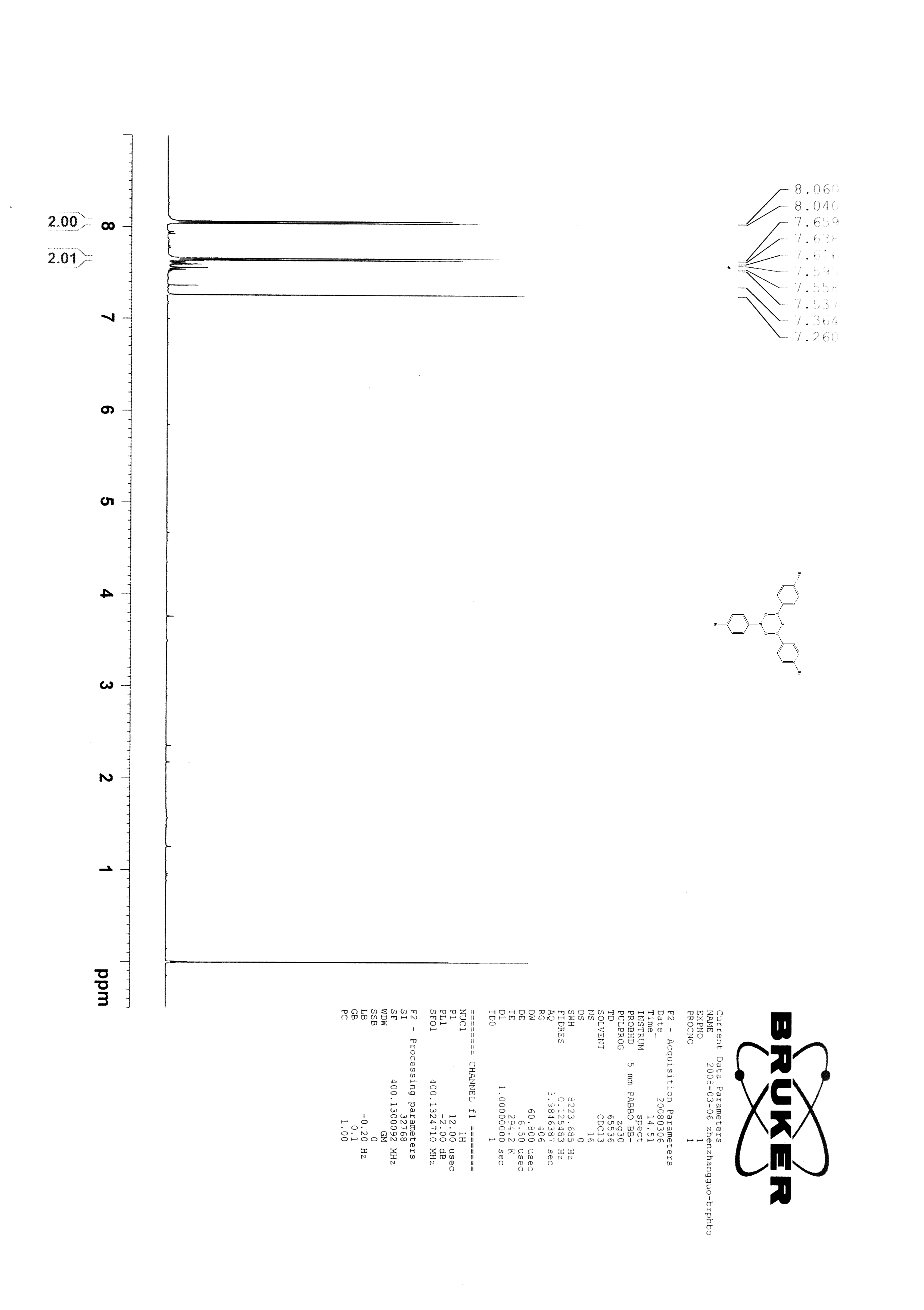
Figure S22 ^1^H NMR of compound **3b**
